# Functional expression of TLR5 of different vertebrate species and diversification in intestinal pathogen recognition

**DOI:** 10.1038/s41598-018-29371-0

**Published:** 2018-07-26

**Authors:** Eugenia Faber, Karsten Tedin, Yvonne Speidel, Melanie M. Brinkmann, Christine Josenhans

**Affiliations:** 1 Medizinische Hochschule Hannover, Institute for Medical Microbiology, Carl-Neuberg-Strasse 1, 30625 Hannover, Germany; 2 DZIF-German Center for Infection Research, Partner site Hannover-Braunschweig, Hannover, Germany; 3 Institute of Microbiology and Epizootics, Free University Berlin, Robert-von-Ostertag-Strasse 7-13, 14163 Berlin, Germany; 4 Helmholtz Center for Infection Research, Inhoffenstrasse 7, 38124 Braunschweig, Germany; 5 Max von Pettenkofer Institute, Ludwig Maximilians University Munich, Pettenkoferstrasse 9a, 80336 Munich, Germany

## Abstract

Toll-like receptor 5 (TLR5) is activated by bacterial flagellins and plays a crucial role in the first-line defence against pathogenic bacteria and in immune homeostasis, and is highly conserved in vertebrate species. However, little comparative information is available on TLR5 functionality. In this study, we compared TLR5 activation using full-length and chimeric TLR5 of various vertebrate species (human, chicken, mouse, pig, cattle). Chimeric TLR5 receptors, consisting of human transmembrane and intracellular domains, linked to extracellular domains of animal origin, were generated and expressed. The comparison of chimeric TLR5s and their full-length counterparts revealed significant functional disparities. While porcine and chicken full-length TLR5s showed a strongly reduced functionality in human cells, all chimeric receptors were functional when challenged with TLR5 ligand *Salmonella* FliC. Using chimeric receptors as a tool allowed for the identification of ectodomain-dependent activation potential and partially host species-specific differences in response to various enteric bacterial strains and their purified flagellins. We conclude that both the extra- and intracellular determinants of TLR5 receptors are crucial for compatibility with the species expression background and hence for proper receptor functionality. TLR5 receptors with a common intracellular domain provide a useful system to investigate bacteria- and host-specific differences in receptor activation.

## Introduction

Toll-like receptor 5 (TLR5) is a crucial determinant of pathogen-host interaction and essential for immune homeostasis^1–4^. Bacterial flagellins of diverse bacteria are the molecular stimuli that ligate and activate TLR5 in various vertebrates^5–9^. TLR5 recognition of bacteria also contributes to non-infectious disease. In particular in the intestinal tract of vertebrates, TLR5 mediates various functions such as shaping the microbiota and immune balance as well as contributing to metabolic tolerance^4,10^. Some bacterial species avoid TLR5 recognition by changing their flagellin protein primary sequence and by structural diversification^11–14^. These evolutionary adaptations might benefit their lifestyle as chronic pathogens, environmental colonizers or symbionts.

In general, the recognition of TLR5 ligands is followed by TLR5 receptor dimerization and subsequent interaction of their intracellular Toll-interleukin-1 receptor (TIR) domains with TIR domains of adaptor proteins, Myeloid Differentiation primary response protein 88 (MyD88) and TIR-domain-containing adapter-inducing interferon-β (TRIF)^15^, leading to the activation of host cell signaling pathways^16,17^. The MyD88-dependent intracellular signaling cascade includes activation of mitogen-activated protein (MAP) kinases and NF-κB, leading to transcription and secretion of proinflammatory cytokines^7,18–21^. Feedback modulation of the signaling cascade after initial activation also leads to the expression and activation of inhibitory molecules of the pathway, such as Toll-interacting protein (Tollip)^22^, the induction of inhibitory miRNAs^23^ and to the degradation of Interleukin-1 receptor-associated kinase 1 (IRAK-1)^24^, which, in a secondary line of signaling, dampens the proinflammatory response (for review:^25^).

Previous studies have addressed the question of species-specific recognition of bacterial flagellins by different vertebrate TLR5^5,26–29^. These approaches mostly relied on heterologous expression systems, where different, mainly full-length TLR5 receptor variants were expressed in human cells or in stably transfected NF-ĸB reporter cell lines. These prior studies have produced conflicting conclusions concerning the activation potential of TLR5 from different species, such as TLR5 of bovine origin^29–31^. It has thus far remained unclear which requirements have to be met for heterologous TLR5 in human cells to be properly expressed, localized and able to signal. Likewise, the use of chimeric Toll-like receptors including TLR5 in human cells^5,32–35^ has been restricted to few studies and has not yet been fully able to clarify the basis of signal transduction by flagellins and other TLR ligands, which is needed to address the question of specific signal uptake via the TLR5 extracellular domain (ECD).

To address some of these open questions, we have expressed and functionally tested TLR5 from various vertebrate species in human cells, either as heterologous full-length receptors or as chimeric receptors, consisting of intracellular (C-terminal) and transmembrane domain of human TLR5, fused to the extracellular (N-terminal) domain of animal origin (chicken, murine, porcine and bovine). The results of our study clarify some of the requirements necessary for the expression and functionality of these heterologous TLR5 receptors. Furthermore, we have used the newly established systems to compare the activation potential of the diverse TLR5 ectodomains in response to the intestinal pathogenic bacterial species *Salmonella enterica* and *Campylobacter jejuni* and their corresponding purified flagellins.

## Results

### Cloning and expression of TLR5 receptors of different vertebrate species in human cells

**Figure 1:**
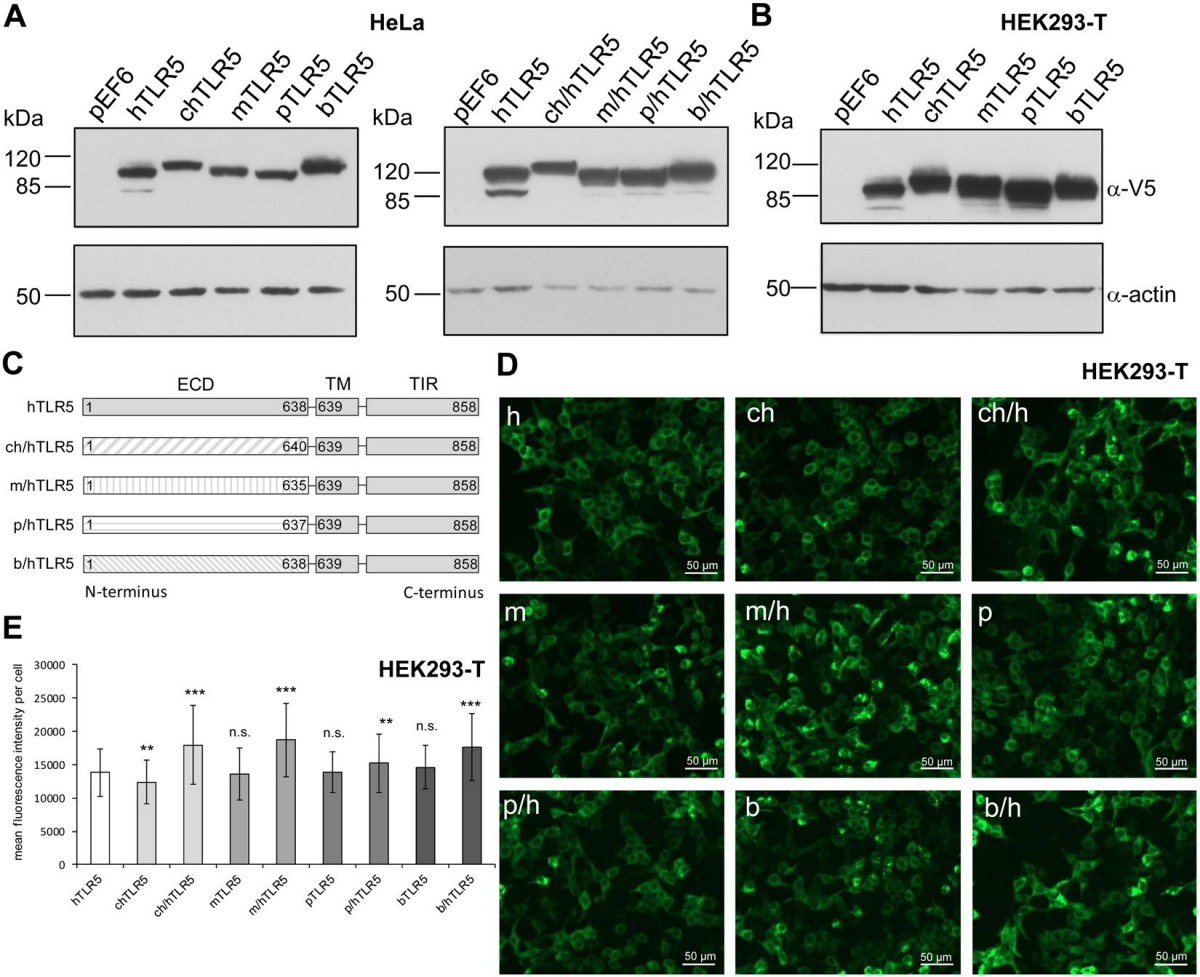
Expression of TLR5 in transiently transfected human cells is similar between TLR5 from different species. HeLa (**A**) or HEK293-T cells (**B**) were transiently transfected with adjusted amounts of plasmids per well coding for TLR5 receptors from different vertebrate species or for chimeric receptors (empty vector pEF6-V5: 200 ng, human (h) TLR5-V5: 100 ng for A or 200 ng for B, chicken (ch) TLR5-V5: 200 ng, chicken/human (ch/h) TLR5-V5: 100 ng, mouse (m) TLR5-V5: 100 ng for A and 200 ng for B, mouse/human (m/h) TLR5-V5: 100 ng, porcine (p) TLR5-V5: 200 ng, porcine/human (p/h) TLR5-V5: 100 ng, bovine (b) TLR5-V5: 200 ng, bovine/human (b/h) TLR5-V5: 200 ng). 48 h post transfection, cells were harvested and cleared lysates analyzed for TLR5 expression using anti-V5 antibody (**A**,**B**). Each construct was tested in at least three independent experiments. (**C**) Schematic depiction of chimeric TLR5 constructs aligned with hTLR5. ECD = extracellular domain. TM = transmembrane domain. TIR = Toll-interleukin-1 receptor domain. Numbers above the boxes designate the amino acids of respective TLR5. (**D**) HEK293-T cells were transiently transfected with human, chicken, murine, porcine, bovine and chimeric TLR5-V5 expression constructs (amounts see above), fixed, labelled with anti-V5 and anti-mouse Alexa Fluor^TM^-488 antibody and analyzed by the automated microscope device Cytation 3 (Biotek). (**E**) Mean immunofluorescence intensity per cell, automatically determined and calculated for every construct; for each condition, 124 cells were measured. Significant differences between hTLR5 and other constructs are indicated by asterisks (Student’s *t*-test, unpaired, two-tailed) as follows: **0.001 < p < 0.01; and ***p < 0.001, n.s. non-significant. The following numbers refer to the determination of transfection efficiencies (ratio of V5-positive divided by DAPI-positive[all] cells, in percent) of the automatic detection system: hTLR5 = 57.44%; chTLR5 = 55.5%; ch/hTLR5 = 73.5%; mTLR5 = 67.76%; m/hTLR5 = 67.74%; pTLR5 = 50.72%; p/hTLR5 = 54.46%; bTLR5 = 47.61%; b/hTLR5 = 66.12%. Full-length and chimeric receptors of the same species show transfection efficiencies in a similar range.

As a prerequisite for testing the activity of diverse vertebrate TLR5 receptors, we cloned the avian (chicken, *Gallus gallus*, chTLR5), murine (*Mus musculus*, mTLR5) and porcine (*Sus scrofa*, pTLR5) TLR5 de novo from cDNAs of respective cell lines (Tables S2, S3). The bovine (*Bos taurus*) TLR5 (bTLR5) was subcloned from a commercially available expression construct (see Methods) into the same expression vector pEF6-V5^14^, used for the other TLR5 constructs (Table S2), for functional comparison with TLR5 receptors from other species. The functionality of the human TLR5 (hTLR5) cloned in pEF6-V5 has been verified previously^9,14^. All TLR5 variants were equipped with a C-terminally fused V5 epitope tag for comparative detection by immunostaining. We transiently transfected human cell lines with the full-length TLR5 constructs, carrying their original N-terminal signal peptides, and verified the expression of all receptor variants by immunoblotting. All TLR5 constructs were detected at the calculated molecular masses in the blots and were expressed to comparable amounts, both in human HEK293-T and HeLa cells (Fig. 1A,B). We then detected the localization of the expressed TLR5 variants in human cells *in situ*, after immuno-labeling of the common C-terminal V5 tag of all TLR5 variants, also using the specific anti-V5-epitope antibody. The transfected cells were pre-screened for efficient fluorescent labeling by manual microscopy and then subjected to automated microscopy and immunofluorescence-based automated image acquisition and quantification of TLR5 expression. The subcellular distribution pattern of the different full-length vertebrate TLR5 receptors in human cells was similar (Fig. 1D). In addition, the automated intensity quantitation and averaging of more than 100 TLR5-positive cells per construct determined that all full-length TLR5 receptors are expressed in similar amounts (Fig. 1E). These findings appeared to provide a suitable basis to perform comparative functional assays on the various full-length TLR5 proteins in the same cellular background.

### Functional testing of full-length TLR5 variants reveals reduced functionality of chicken and porcine TLR5 in human cells

**Figure 2:**
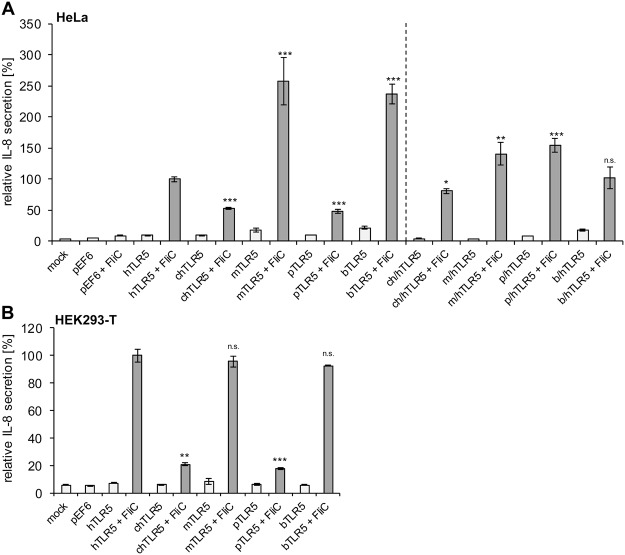
Activation deficiency of chicken and porcine TLR5 can be partially rescued with respective TLR5 chimeras. HeLa (**A**) or HEK293-T cells (**B**) were transiently transfected with adjusted amounts of plasmid DNA encoding for TLR5 from different species or chimeric receptors (transfected plasmid amounts see Fig. 1). 48 h after transfection, cells were coincubated with purified recombinant *Salmonella* FliC (50 ng/well) or mock-treated for four hours. IL-8 secretion in the cell supernatants was determined by ELISA. IL-8 secretion of hTLR5-transfected, FliC-stimulated cells was set to 100% (reference); relative IL-8 secretion of all constructs with regard to the reference is depicted. Each condition was tested in two independent biological replicates (each in technical triplicates), the results of which are summarized here as mean and standard error. One representative experiment out of three is shown. For all constructs in A and B, except for pEF6-empty, the differences between mock-coincubated and FliC-coincubated condition were highly significant (Student’s *t*-test, unpaired, two-tailed; p < 0.01). Likewise, all differences between the full-length and respective chimeric constructs (in A) in the FliC-coincubated condition were also highly significant (p < 0.01). For clarity, asterisks here indicate solely the significant differences between FliC-activated hTLR5 and the corresponding activated full-length or chimeric constructs (Student’s *t*-test, unpaired, two-tailed) as follows: *0.01 < p < 0.05; **0.001 < p < 0.01; and ***p < 0.001, n.s. non-significant.

In order to assess the activation potential of all expressed full-length TLR5 from different species, we next performed cytokine measurements in supernatants of non-activated versus flagellin(FliC)-activated cells (see Fig. S1, for purification of recombinant FliC flagellin). Various vertebrate TLR5 receptors showed differential activities towards the canonical ligand *Salmonella* FliC (Fig. 2). Mouse and bovine full-length TLR5 were highly activated, both in HEK293-T and in HeLa cells, to induce IL-8 secretion in the human cellular background, in HeLa cells even significantly higher than the full-length human TLR5 (Fig. 2A). Chicken and porcine TLR5 receptors displayed significantly lower activation levels (IL-8 release) in HeLa (Fig. 2A) and HEK293-T (Fig. 2B) cells when compared to the other receptors and to full-length human TLR5, although they still showed a significant increase of IL-8 by FliC (Fig. 2B). Reduced activation potency of the chicken and porcine receptors for IL-8 secretion was more pronounced in HEK293-T in comparison to HeLa cells. When tested in control experiments, differential expression levels of TLR downstream signaling components MyD88, IRAK-1, and IRAK-M were clearly detectable between the two human cell lines (Fig. S2). For instance, MyD88 was much higher expressed in HeLa cells. These clear-cut cell-specific characteristics provided a likely cause for the observed cell-specific activation differences, in particular the higher activation in the HeLa cells by chicken and porcine TLR5. Other control experiments excluded nucleotide differences in the promoter region, in the Kozak sites introduced by the cloning procedures, or an interference by the C-terminal V5 tag as being responsible for the differential activation potential of the full-length chicken TLR5 (Fig. S3). We therefore considered that potential species barriers between the non-human receptors (chicken and porcine) and the human cells are one possible explanation for the observed phenotype of their decreased function. For instance, diversity in the intracellular TIR domain structure or the adjacent transmembrane segment of the respective TLR5 species variant may mediate incompatibilities to downstream adaptor proteins such as MyD88 in human cells.

### The expression of TLR5 chimeras in human cells partially restored the functionality of chicken and porcine TLR5 ectodomain chimeras for IL-8 secretion and NF-κB activation

In order to address the question of such potential species barriers experimentally, we designed equivalent TLR5 chimeras of all previously tested TLR5 variants. These chimeric receptor constructs consist of the ectodomain (ECD) sequences of the diverse vertebrate TLR5 (chicken, mouse, porcine and bovine), fused in-frame with the sequences for transmembrane segments and intracellular domain (ICD) of human TLR5 (Figs 1C, S4). All TLR5 chimeras were efficiently and comparably expressed after transient transfection, as verified by Western blotting and immunofluorescence-based automated quantification (Fig. 1A,E). Also, their expression patterns in cells *in situ* were again similar when compared to the full-length TLR5 of all tested species. The downstream signaling potential of the TLR5 chimeras was further verified and compared to the full length receptors by measuring IL-8 release. All chimeric receptors activated human cells efficiently and significantly to secrete IL-8 upon stimulation with *Salmonella* FliC flagellin (Fig. 2A). Therefore, we anticipated that these heterologous TLR5 ectodomain constructs, in contrast to the full length receptors, would be better suited to conduct comparative activation studies within one common cellular background.

**Figure 3:**
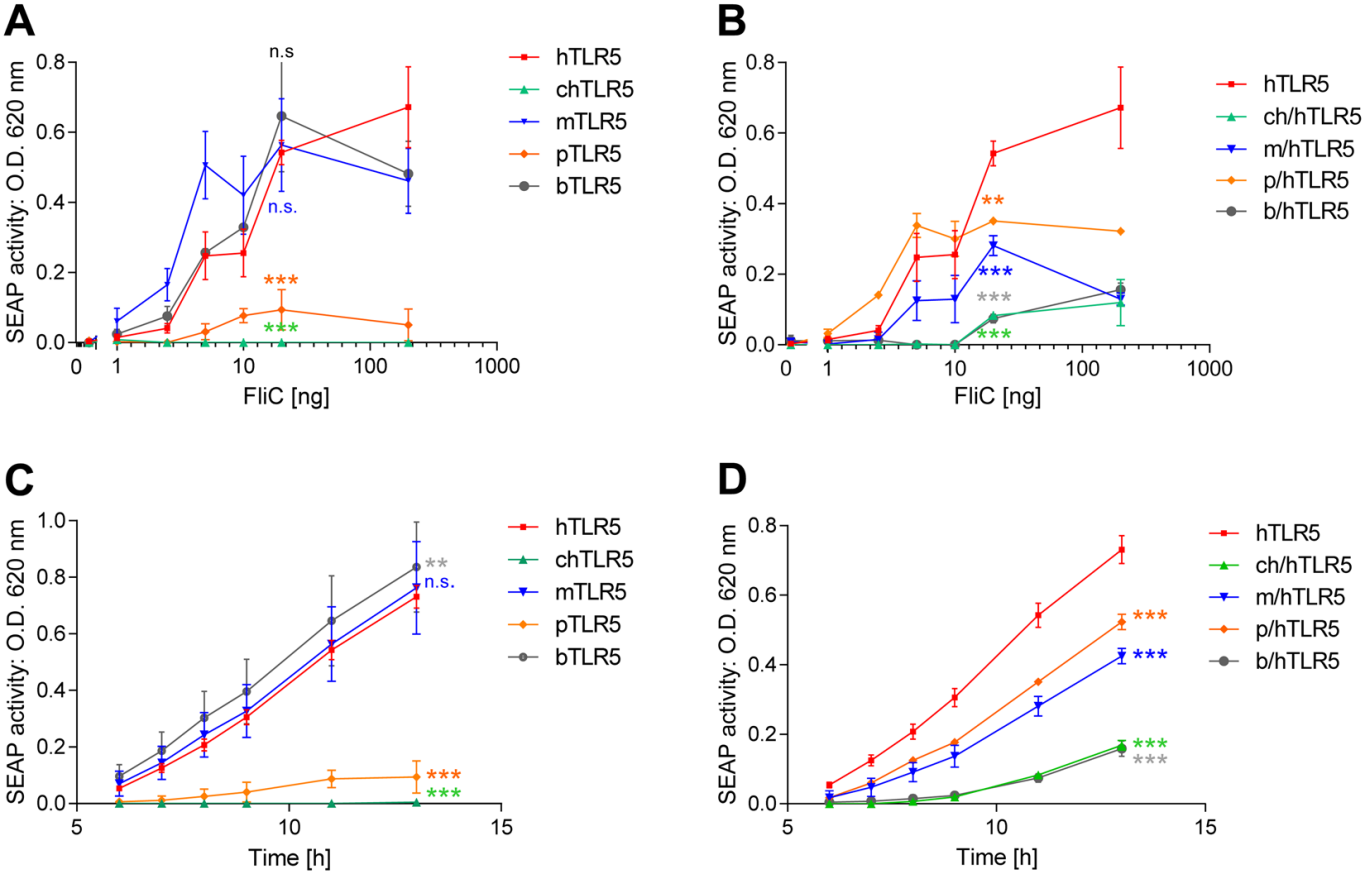
Concentration dependency and time course of activation of NF-ĸB reporter cells expressing heterologous and chimeric TLR5 receptors from different vertebrate species by *Salmonella* FliC. HEK-Blue Null1 reporter cells were transiently transfected with adjusted amounts of plasmid DNA per well, encoding for full-length TLR5-V5 from different species, or with chimeric receptor constructs (empty vector pEF6-V5: 100 ng; human (h): 50 ng; chicken (ch): 50 ng; chicken/human (ch/h): 50 ng; mouse (m): 100 ng; mouse/human (m/h): 50 ng; porcine (p): 100 ng; porcine/human (p/h): 100 ng; bovine (b): 100 ng; bovine/human (b/h): 100 ng; 96-well format). 24 h post transfection, the cells were coincubated with different amounts of purified recombinant *Salmonella* FliC (1 ng to 200 ng per well) over a time course of 13 h. NF-κB-dependent SEAP production by the reporter cells was determined by colorimetric measurements at 620 nm (see Methods) and served to determine the optimal concentration and time parameters for further coincubation assays. Panels (A,B) show concentration-dependent activation of all non-chimeric (**A**) and chimeric (**B**) constructs. Panels (C and D) show time-dependent activation of all non-chimeric (**C**) and chimeric (**D**) constructs, all coincubated with 10 ng FliC ligand per well. Mean and standard deviation from technical triplicates of a representative experiment are shown. Significant differences between FliC-activated hTLR5 and the corresponding non-chimeric and chimeric activated constructs are indicated by asterisks (multiple comparison by two-way ANOVA, Dunnett’s multiple comparison test of activation by 20 ng FliC in (**A**,**B**) and for the whole activation time course in (**C**,**D**) as follows: *0.01 < p < 0.05; **0.001 < p < 0.01; and ***p < 0.001, n.s. non-significant.

To strengthen the basis for further functional assays, we quantitated in more detail the activation potential of all full-length TLR5 and all chimeric receptors. For this purpose, we performed NF-κB reporter assays with recombinant *Salmonella* FliC flagellin as reference ligand, both in a concentration-dependent manner (Fig. 3A,B), and during a time course (Fig. 3C,D), using HEK-Blue Null1 reporter cells, which allow high-throughput screening for NF-κB activation. The results of these experiments confirmed the reduced activation potential, in a concentration-independent manner, for full-length porcine TLR5 and full-length chicken TLR5 (Fig. 3A) at the level of NF-κB. The porcine/human (p/h) TLR5 chimera recovered significantly elevated NF-κB activation in a wide FliC concentration range (Fig. 3B). The chicken/human (ch/h) chimera recovered some (significant) activation potential, however only at higher FliC ligand concentrations (>10 ng FliC per well in a 96-well format) (Fig. 3B). During the time course experiment, the full-length chicken TLR5 did not show increased NF-κB activation after longer coincubation times with a fixed amount of FliC, while the porcine full-length TLR5 showed some, low-level, activation (significant) at later time points. Time-dependent NF-κB activation was restored for the ch/h and p/h TLR5 chimeras, although the maximal activation levels of those two chimeras in this setting still remained significantly lower than for the full-length human TLR5 (Fig. 3D). Bovine and mouse TLR5 chimeras showed a reduced NF-κB activation potential (maximum of absolute reporter induction values) in comparison to the respective full length receptors, for the dose-response experiment and during the time course. However, FliC-coincubated b/hTLR5 and m/hTLR5 still provided markedly elevated (highly significant) NF-κB activation in comparison to the mock-coincubated condition (Fig. 3B,D).

### Functionality and intracellular signaling events of heterologous full-length TLR5 and TLR5 chimeras in human cells

**Figure 4:**
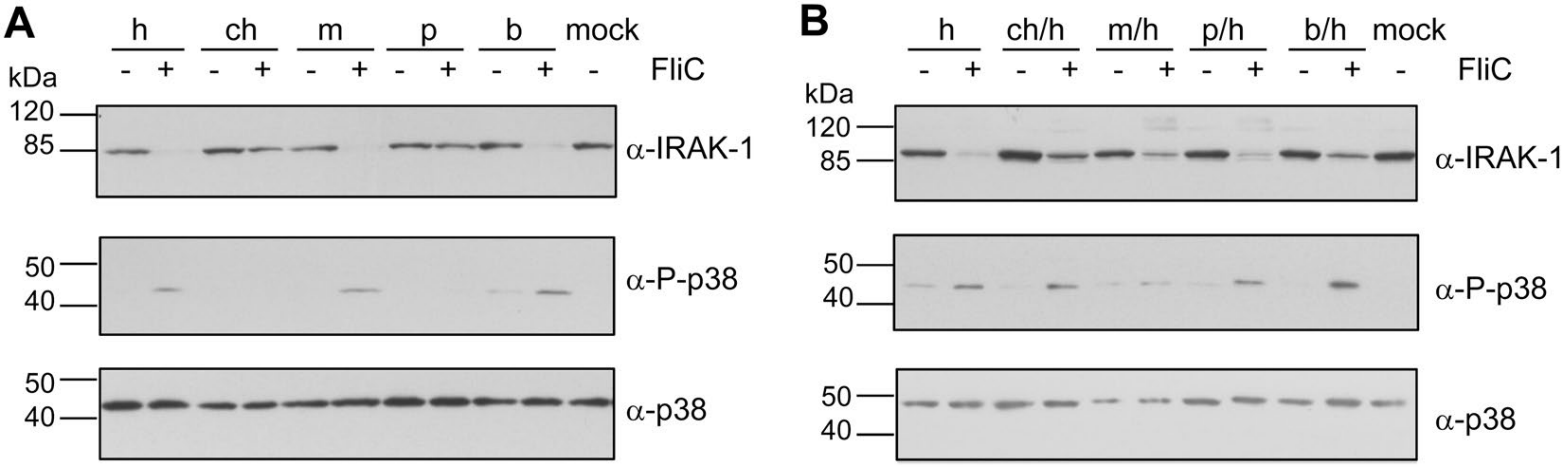
Comparative analysis of TLR downstream signaling after heterologous TLR5 activation indicates major contribution of intracellular domain in signaling deficiencies. HeLa cells were transiently transfected with indicated TLR5-V5 plasmids (transfected plasmid amounts as in Fig. 1) and subsequently activated with recombinant *Salmonella* FliC (50 ng/well) for four hours. Cleared cell lysates were analyzed by immunoblotting for posttranslational modification of p38 and degradation of IRAK-1. Phosphorylation (=activation) of p38 was visualized by a phospho-specific p38 antibody (α-P-p38) and is shown in comparison to the same blot probed with α-p38 antibody. Degradation of human IRAK-1 triggered TLR5 signaling was detected by an α-IRAK-1 antibody (Table S6 for antibodies).

All chimeric and non-chimeric constructs were compared with regard to different branches and downstream events of TLR5 signaling, namely p38 activation by phosphorylation and the degradation of IRAK-1, which is a feedback mechanism after successful docking of the TLR5 TIR domain to downstream adaptors^36^. As shown in Fig. 4, chicken and porcine TLR5 were clearly comfirmed to have a functional deficit in human cells, since they activated p38 poorly and did not lead to the degradation of IRAK-1 (Fig. 4A) upon stimulation by *Salmonella* FliC (50 ng/well) for four hours. In contrast, all TLR5 chimeras activated both downstream pathways (Fig. 4B). In order to identify a potential molecular basis for the species barrier between chicken and human TLR5 receptors, respectively, one point mutation was introduced into full-length chicken TLR5 at amino acid position 746 in the C-terminal domain (R746 in chicken and Q744 in human TLR5) close to the BB loop, which has been shown to interact with downstream TIR domains of human MyD88 (Fig. S5). This residue is one of the few in this well-characterized segment of TLR5 receptors which is divergent between the heterologous TLR5 variants and human TLR5. This residue in full-length chicken TLR5 was changed to the human-specific amino acid, resulting in the site-mutated construct chTLR5(R746Q). Interestingly, this point-mutated TLR5 variant was not expressed in detectable amounts in cleared lysates of transiently transfected human cells and did not recover functionality (Fig. S5). The lack of expression was accompanied by a massive phosphorylation of p38, suggesting cellular endosomal stress activation, possibly due to a trafficking impairment that can lead to downstream stress signaling^37^. Two sequence polymorphisms in the ectodomain of the cloned full-length chicken TLR5 and the ch/h chimera were identified by Sanger sequencing in comparison to the reference sequence in the NCBI database (NP_001019757.1) (Suppl. Fig. S11). These exchanges were non-synonymous (aa 29D to V and aa 90 S to G), and localized far from the leucine-rich repeat 9 (LLR9) which directly interacts with the flagellin ligand^17^. For full-length porcine TLR5, complete plasmid sequencing verified that the coding region entirely matched the respective reference sequence (NP_001335700.1).

### Reciprocal expression of selected TLR5 constructs in chicken cells verifies that human and chicken TLR5 are functional in chicken cell**s**

**Figure 5:**
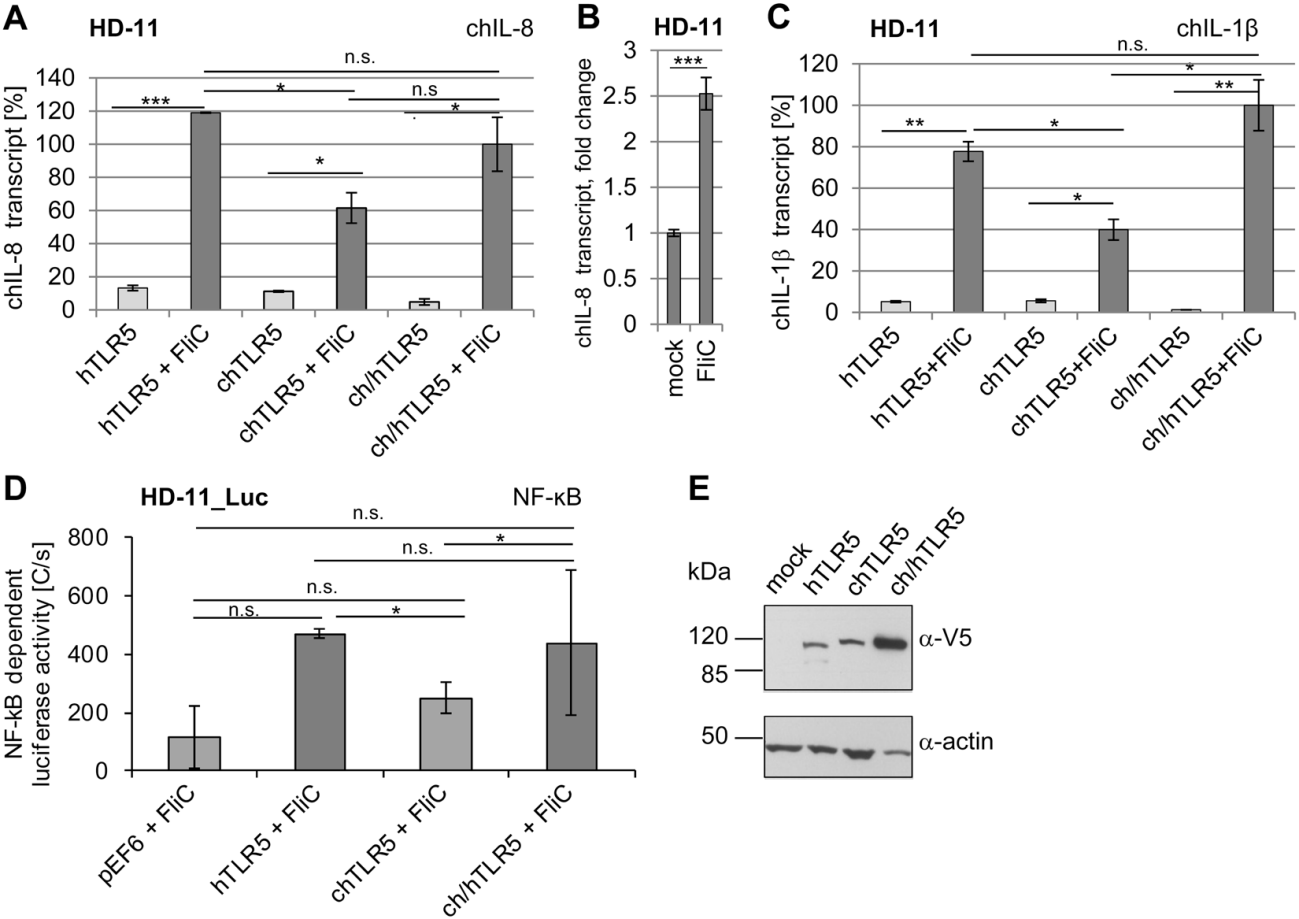
Reciprocal expression and functionality of human, chicken and chimeric TLR5 within chicken cellular background. Chicken HD-11 (**A**,**B**,**C** and **E**) or NF-κB reporter HD-11 cells (**D**) were nucleofected with expression plasmids coding for human (h), chicken (ch) or chimeric (ch/h) TLR5 from different species as indicated in the Methods. 24 h post nucleofection, selected wells were coincubated with purified recombinant *Salmonella* FliC (200 ng/well in 6-well plate format or 25 ng FliC in 96-well plate format). For quantitative RT-PCR, cells were stimulated for 30 min, and transcript amounts of chicken IL-8 (**A**,**B**) and IL-1β (**C**) were determined. Quantitated transcript values were normalized to respective chicken GAPDH transcript amounts and are presented in [%] relative to FliC-activated ch/hTLR5, set to 100%. In panel B, chIL-8 transcript induction in FliC-activated versus mock-coincubated non-transfected HD-11 cells is shown as fold induction. This result demonstrates a significant but considerably lower response of intrinsic chTLR5 in comparison to the activities provided by transfected TLR5 expression constructs shown in **A** and **C**. NF-κB-dependent luciferase activity was measured after 3.5 h of HD-11_luc stimulation using SteadyGlo luciferase assay (**D**). In D, the respective control values for all constructs (pEF6-V5 empty, hTLR5, chTLR5, ch/hTLR5) without FliC activation were subtracted from each FliC-activated value as background to yield the depicted activated end values for each construct in luminescence photon counts [C per s]. Mean and standard deviation from technical duplicates for transcriptional activation (**A**,**C**) and from technical triplicates for intrinsic TLR5 activity in HD-11 (**B**) and NF-κB-dependent activation (**D**) are shown. All experiments were independently repeated at least once, with similar outcomes. Significant differences between specific conditions are indicated by lines and asterisks in A, B, C and D (Student’s *t*-test; *0.01 < p < 0.05; ***p < 0.001; n.s non significant). Western Blot analysis for expression of TLR5 constructs using cleared lysates of nucleofected HD-11 cells (**E**). Immunoblotting was performed using anti-V5 or anti-actin antibody (loading control).

In order to analyze chicken TLR5 functionality in more detail, since we suspected a potential species barrier, a reciprocal experimental approach was performed. The chicken macrophage-like cell line HD-11 or a stably transfected NF-κB HD-11 luciferase reporter line (HD-11_Luc^38^) were used for transient transfection with chicken TLR5, human TLR5 and the corresponding TLR5 chimera via nucleofection. Both human and chicken TLR5 were functional in the chicken cellular background, and activation with recombinant *Salmonella* FliC for two hours resulted in significantly enhanced transcript levels of chicken IL-8 and IL-1β cytokine genes (Fig. 5A,C) and stimulation of NF-κB (Fig. 5D). Moreover, expression and activation were comparable between the chicken and human full-length TLR5 constructs in HD-11 cells, demonstrating compatibility of human TLR5 with chicken cells (Fig. 5A,C and E). Of note, nucleofection of HD-11_luc cells with chimeric TLR5 (chicken-human) resulted in a high background activation of NF-κB and enhanced cell death two days after nucleofection. Taken together, the reciprocal experiment suggests that chicken cells can, in principle, support, the activity of full-length human TLR5, while this is less the case vice versa. It cannot be excluded at present that this differential species-specific phenotype is associated with the respective cell or cell type (epithelial versus macrophage).

### Various TLR5 chimeras are differentially activated by *Salmonella enterica* serovars or their purified flagellins

**Figure 6:**
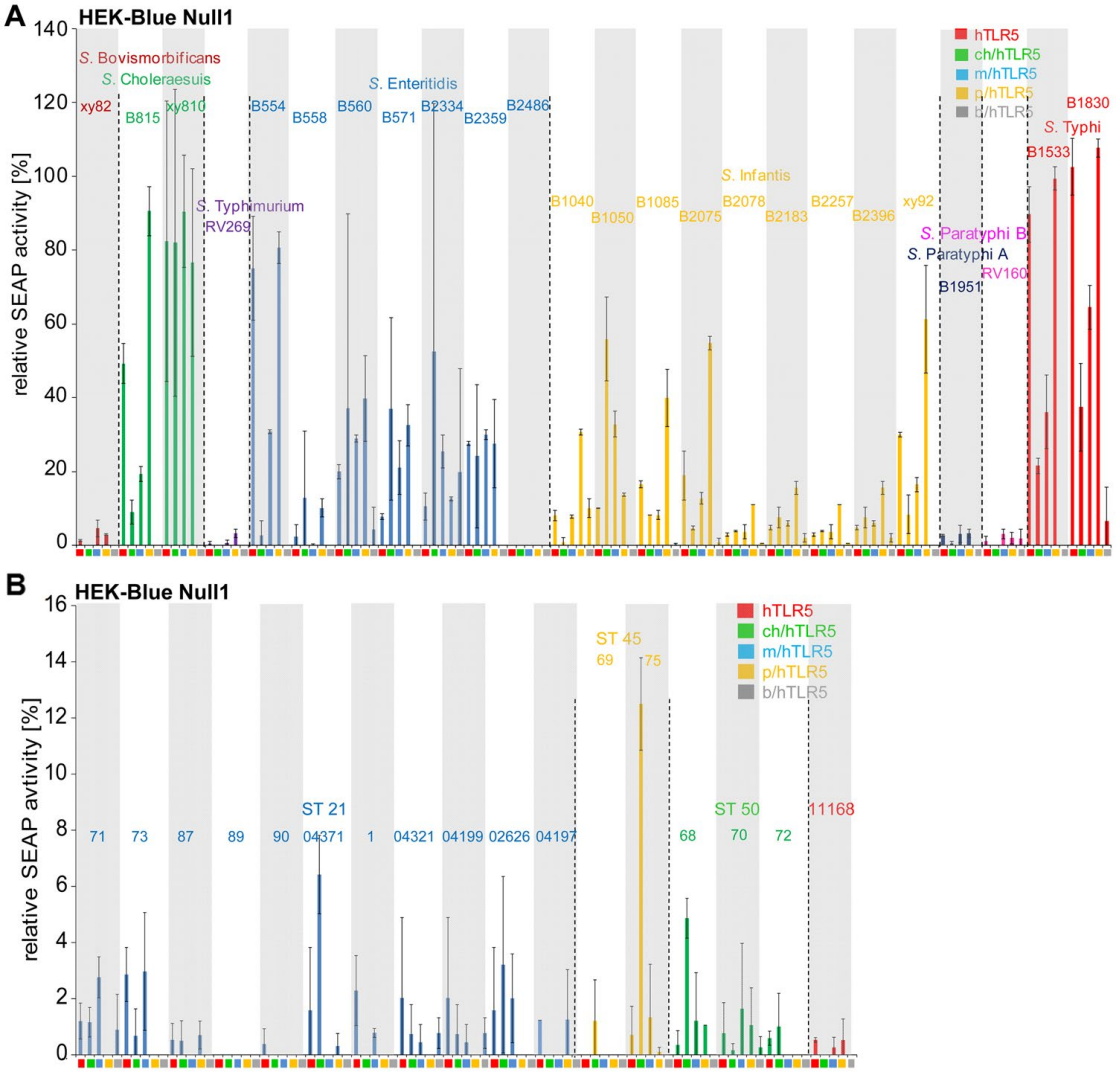
Comparison between activation potential by lysates of various *Salmonella enterica* serovars (**A**) and *Campylobacter jejuni* (**B**) strains on TLR5 chimera-expressing NF-ĸB reporter cells. HEK-Blue Null1 NF-κB reporter cells were transiently transfected with appropriate amounts of expression plasmids coding for human TLR5-V5 or for chimeric receptors (empty vector pEF6-V5: 100 ng, human (h): 50 ng, chicken/human (ch/h): 50 ng, mouse/human (m/h): 50 ng, porcine/human (p/h): 100 ng, bovine/human (b/h): 100 ng). The different TLR5 chimeric constructs are color-coded as indicated below the x-axis and in the graph. After 24 hours, transfected cells were coincubated with *Salmonella enterica* or *C. jejuni* lysates (100 ng total protein content per well) for 11 h; NF-κB-dependent SEAP production was determined by colorimetric measurements at 620 nm (see Methods). Values are depicted as relative values (in percent) to each corresponding construct activated by 20 ng of recombinant FliC as reference, which was set to 100%. Empty vector-transfected cells activated by lysate were defined as background and subtracted for each strain separately. This evaluation does not allow a direct comparison between chimeras, but allows the direct comparison between different bacterial strains/serovars for each chimeric receptor, and of relative host activation patterns between strains. Activation by single strains of each serovar is separately highlighted by alternating white or grey background shading. Additionally, strain groups are subdivided according to their serovar or sequence type (ST, for *C. jejuni* isolates) by dashed lines; each serovar or strain group (ST) and the group-specific strain names (according to Table S1) are indicated above the bars and color-coded in the same color. Mean and standard deviation from biological triplicates of a representative experiment (out of two independent experiments) are shown. For *C. jejuni*, results for strain 11168 are given as a reference.

The constructed TLR5 chimeras offered the possibility to compare activation potential mostly dependent on the ECDs of TLR5 from different vertebrate species in response to flagellins of bacterial pathogens with different host adaptation phenotypes. Applying our chimeric test system, we next focussed on the investigation of TLR5 activation by the important human enteric pathogens *Salmonella enterica* (different serovars; Supplementary Table S1) and preselected, variable *Campylobacter jejuni* strains (Table S1). We compared ECD-dependent activation potential of various respective serovars (*S. enterica*) or strains (*C. jejuni*) on human (full length TLR5), or chicken, mouse, porcine and bovine TLR5 chimeras. With this aim, a reporter cell line (HEK-Blue Null1 SEAP reporter cells) for NF-κB-dependent activation, which is suitable for screening numerous ligands simultaneously in real time, was transiently transfected with full-length human or chimeric TLR5 constructs and activated using sonicated, homogenized bacterial lysates (Fig. 6). Interestingly, lysates of 24 different *S. enterica* isolates showed a differential, serovar-dependent activation potential in this system. Lysates of *Salmonella* serovars Choleraesuis and Typhi were highly activating stimuli on all TLR5 chimeras compared to the reference FliC, whereas lysates of *S. enterica* serovars Enteritidis and Infantis showed an overall lower, intermediate, activating phenotype in comparison to the reference. Isolates of *S. enterica* serovars Bovismorbificans, Typhimurium, Paratyphi A and Paratyphi B exhibited a comparable and very low activation potential in this setting using 100 ng of bacterial lysate per well. A higher activation by those latter *Salmonella* lysates was found to be concentration-dependent. This was confirmed for the activation of hTLR5 by various amounts (500 ng to 3 µg) of *S*. Typhimurium lysate (Fig. S6B). For the single chimeras, TLR5 receptors with human and porcine ECDs resulted in higher activation levels with all of the analyzed *Salmonella* lysates, followed by mouse and chicken chimeric receptors in the order of decreasing activation. Relative NF-κB activation of the bovine chimera was very low for the chosen lysate amounts with all tested *Salmonella* lysates in comparison to purified recombinant *Salmonella* FliC used as a reference.

**Figure 7:**
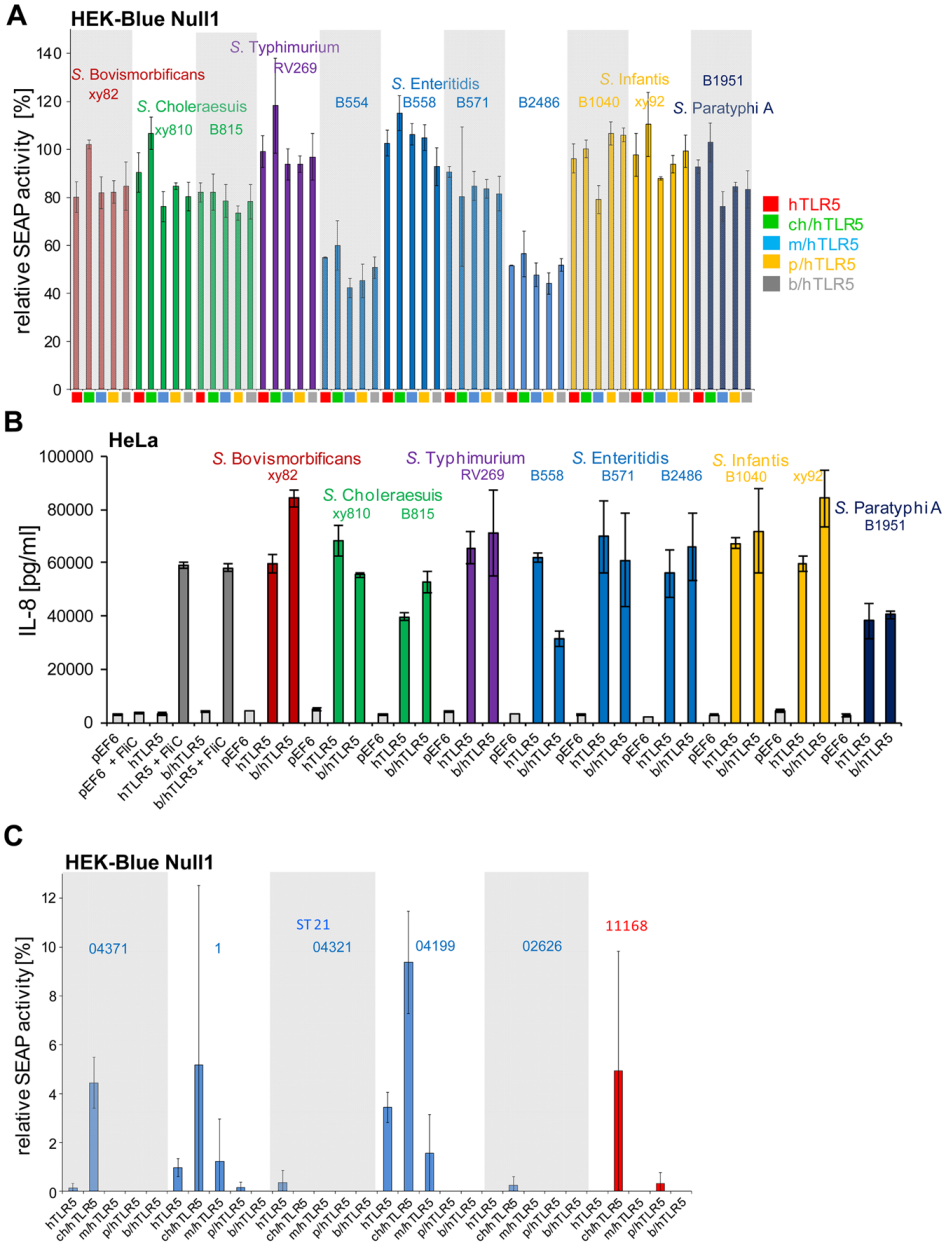
Comparison between activation potential by flagellar fractions of selected *Salmonella* serovars (**A**,**B**) and *C. jejuni* isolates (**C**) on chimeric TLR5 receptors. (**A**) HEK-Blue Null1 NF-κB reporter cells were transiently transfected with appropriate amounts of expression plasmids coding for human TLR5-V5 or for chimeric receptors (empty vector pEF6-V5: 100 ng, human (h): 50 ng, chicken/human (ch/h): 50 ng, mouse/human (m/h): 50 ng, porcine/human (p/h): 100 ng, bovine/human (b/h): 100 ng). After 24 hours, transfected SEAP reporter cells were coincubated with *Salmonella* flagellar protein-enriched fractions (25 ng protein per well) for 11 h; NF-κB-dependent SEAP production was determined by colorimetric measurements at 620 nm (see Methods). Values are depicted relative to the activation of the corresponding construct stimulated by 20 ng of recombinant *Salmonella* FliC as reference, which was set to 100%. Background of empty vector-transfected, flagellin-activated cells was subtracted. (**B**) IL-8 release of HeLa cells (absolute values in pg per ml) transfected for human TLR5 and bovine-human TLR5 chimera was determined after 4 h of coincubation with *Salmonella* flagellin-enriched fractions (125 ng protein) or recombinant FliC as positive control (50 ng/well; grey bars) of cells transiently transfected for 48 h. For each condition, mean and standard error of biological duplicates measured in technical triplicates are shown. The experiment was independently repeated once with similar results. In (**A**,**B**), flagellins from *Salmonella* strains B554, 571 and B2486 are representative for FliC-only strains, while B815 and xy92 only express FljB. (**C**) Activation by *C. jejuni* flagellin-enriched fractions of chimeric TLR5-transfected HEK-Blue Null1 cells was determined as described for *Salmonella* flagellin in **A**. For (**A**,**C**), activation by single strains of each serovar is separately highlighted by alternating white or grey background shading. For (**A**,**C**), mean and standard deviation from biological triplicates of a representative experiment are indicated. Each serovar or sequence type (ST, for *C. jejuni* isolates) and names of the group-specific strains (according to Table S1) are indicated above the bars and color-coded in the same color. The different expression constructs are color-coded as indicated below the x-axis and to the right side of the graph (for panel A) or indicated below the x-axis (for panels B and C).

In order to compare the amounts of flagellins in the selected *Salmonella* lysates, we performed immunoblotting of lysates using anti-*E. coli* flagellin antibody. Lysates of the respective different *Salmonella* serovars contained comparable amounts of flagellin (Fig. S7). Only *S*. Enteritidis B 554 and *Salmonella* Typhi lysates exhibited clearly lower flagellin amounts, despite the fact that both showed an overall strong activation with all chimeric TLR5 constructs. Flagellins from eleven selected serovars corresponding to some of the lysate preparations were subsequently purified as native proteins from the respective *Salmonella* strains and used in equalized amounts to activate human cells in the chimeric TLR5-expressing, SEAP-dependent human reporter cell system. In contrast to whole bacterial lysates, all of the purified flagellar fractions activated the tested chimeras to a comparable extent, independently of the analyzed serovar (Fig. 7A). No or only minor differences between the activation potential of the respective flagellar fractions were also confirmed at the level of secreted IL-8 (Fig. 7B). In line with these results, Sanger sequencing of phase one and two flagellins (*flgJ* and *fliC* genes), respectively, from the tested *Salmonella* serovars did not reveal sequence differences at positions known to be important for TLR5 activation (Figs S9 and S10). Some strains only possess either *flgJ* (e.g. B1050) or *fliC* (e.g. B2359) genes, serving as type-specific controls (Figs S9 and S10). Taken together, purified native *Salmonella* flagellins activated TLR5 variants in a rather equivalent manner, while diverse *Salmonella* strain lysates showed heterogeneous activity via TLR5.

### Low extent of cell activation via TLR5 chimeras by various *Campylobacter jejuni* strains from different origins or their isolated flagellins

*C. jejuni* flagellin has been reported to have a low activation capacity on TLR5^11,39,40^. However, this has not been analyzed in a comparative setting for TLR5 from a variety of vertebrate species nor for various different *C. jejuni* strains. In order to analyze species-specific TLR5 activation by *C. jejuni*, bacterial lysates and flagellar fractions were generated and tested comparatively for activation of the TLR5 chimeras for 17 human and animal *C. jejuni* isolates preselected after molecular typing, and including the reference strain 11168. In contrast to the results found with *Salmonella* serovars, the tested *C. jejuni* lysates did not show a significantly enhanced NF-κB activation of the various chimeric TLR5 constructs (Fig. 6B), independently of the applied amounts. Concentration-dependent maintenance of low activation was further tested for *C. jejuni* lysates 73 and 75 (at 500 ng to 3 µg per well) and hTLR5 (Fig. S6B). In addition to the results obtained with bacterial lysates, no significant activation at the level of NF-κB was detected using isolated flagellin-enriched fractions of the tested *Campylobacter* species in the setting of TLR5 chimeras expressed in HEK NF-κB reporter cells (Fig. 7C). To summarize the latter findings, *C. jejuni* flagellins and lysates of various well-characterized isolates, preselected for genetic diversity, were not active on any of the expressed TLR5 chimeras in comparison to the FliC reference, regardless of *C. jejuni* strain origin or molecular sequence type of the respective strains.

## Discussion

Toll-like receptors are key innate immune pattern recognition receptors (PRR) (for reviews see^1,41,42^. They are not only important for defence reactions of cells against invading pathogens, but also contribute to the establishment of an immune homeostasis in conjunction with the resident microbiota at different body sites^3,4,43^ and prevent excessive immune activation^3,44–46^. TLR5 is known to play a major role in mediating both of these tasks, especially with regard to anti-pathogen defence and stable colonization in the intestinal tract^10,44,47^. TLR5 signaling is therefore not only required for proper functionality of the innate arm of the immune system, but moreover contributes to the development of adaptive mechanisms, in order to maintain the balance of motile members of the microbiota, reduce the immunostimulatory load in the gut^44^, and providing temporal stability to the microbiota composition^10^.

Of note, TLR5 is the only PRR so far that recognizes a specific bacterial protein, flagellin, which is the exposed subunit of the bacterial motility organelle, the flagellum^8^. Numerous intestinal bacterial species, pathogens as well as commensals, express flagella and can be recognized by TLR5^5,9,48^. Evasion of TLR5 recognition by specific bacterial flagellins has been described^11,13,14^, which may be of relevance for chronic colonizing bacteria, pathobionts and pathogens of the gastrointestinal tract.

The importance of functional TLR5 for colonization resistance against pathogenic bacteria becomes apparent in humans harboring TLR5 polymorphisms, which predispose to various infectious diseases^49–51^. Some non-synonymous polymorphisms within the coding sequence of human TLR5 affect its signaling capacity and human disease susceptibility^52,53^. Sequence polymorphisms in *tlr5* and associated changes in functionality have been identified within various animal species^54–57^. Several non-synonymous polymorphisms in both the ECD or the ICD were described to attenuate TLR5 responses towards flagellin, demonstrated for both porcine (polymorphisms R148L and P402L)^56^ and canine TLR5 (polymorphisms C100T and T1844C)^55^. In contrast, other amino acid exchanges resulted in a higher activation and have been suggested to be linked to enhanced susceptibility to inflammatory bowel disease, as described for the canine TLR5 (polymorphism G22A)^55^. In passerine birds, a fixation of a non-functional TLR5 variant in combination with several independent pseudogenisation events of TLR5 has been reported^54^. Despite the clear general importance of TLR5, the reported variations in TLR5 functionality at the level of host species populations or individual animal breeds complicate general conclusions about TLR5 signaling for a whole species and require further clarification and comparative approaches. In this context, we have clarified in the present study basic requirements for TLR5 receptor function across various vertebrate species and generated a system to investigate the contribution of TLR5 ECD variants to host- and pathogen-species-specific signal transduction and signal strength.

Several questions remain to be clarified with respect to the role of TLR5 in the immune response in different species. Most genomes of vertebrate species characterized to date contain one TLR5 gene copy^58,59^. Although mouse and human TLR5 have been characterized in some detail^5,27^, and a structure of zebrafish TLR5 dimer ligated with flagellin has been published^17^, detailed mechanisms of TLR5-dependent or -independent signaling by bacterial flagellins are not completely understood. Likewise, the expression, signaling, and role of TLR5 in vertebrate species other than human or mouse are poorly characterized. It is also largely unknown, whether host species-specific traits of TLR5 signaling may exist. In particular, the roles of the receptor transmembrane domains and intracellular domains have not been explicitly studied, including differences in the compatibility of intracellular TLR5 TIR domains with downstream adaptors (e. g. MyD88) between species. It is also insufficiently understood, whether various bacterial species can induce differential TLR5-mediated responses in a strain- or host-specific manner. In the present study, we have therefore examined how TLR5 variants from different host species support signaling in a comparative, heterologous host setting, and developed a cell-based test system to determine host specificity of various vertebrate TLR5 variants. This approach has helped us to elucidate that there are potential incompatibilities at the molecular level, which are determined by both, the receptor ECDs and ICDs, or even by both, ECD and ICD, in combination, that may impair the functionality of some vertebrate TLR5 in human cells. Functional deficiencies of some vertebrate TLR5 (porcine, chicken) in human cells were identified. This prompted us to also establish an expression system using chimeric TLR5 with various vertebrate ECDs fused to the intracellular domain of human TLR5. We validated the functionality of the chimeric test system, which served as an indicator of ECD-dependent ligand recognition, and subsequently compared the activation potential of various *S. enterica* and *C. jejuni* isolates and their flagellins in cells expressing the TLR5 chimeras.

Conflicting evidence has been reported for the functionality of bovine TLR5 variants when expressed in human cells^29–31^. One group observed no functionality of bovine TLR5 within the human HEK293-T cellular background, which could be partially restored by the substitution of phenylalanine to tyrosine at position 798 (aa 798 F to T), suggested to be a putative binding site for phosphatidylinositol 3-kinase^29,30^). One other group successfully activated native bovine TLR5 within human HEK293 cells by H7 flagellin from *E. coli*^31,60^. In our hands, wild-type bovine TLR5, which exhibited phenylalanine at position 798, was active when expressed in human cells (e.g. HEK293-T or HeLa). Differences between the studies might be explained by the different reporter systems used. We conclude from our comprehensive results that native bovine TLR5 (F798) is fully functional in human cells corroborating the latter studies^31,60^. Full-length porcine TLR5 showed functional defects within human cells which could be recovered in the porcine/human chimera. This result strongly suggested that incompatibility of the porcine receptor with the downstream human cellular system is largely restricted to the receptor’s ICD, which underscores the validity of the chimera approach.

Chicken (avian) TLR5 shows the largest deviation in sequence from other TLR5 of various mammals, both in the ECD and ICD domains (Fig. S8). We increased the functionality of the chicken TLR5 ECD in human cells by fusion to the human TLR5 intracellular TIR domain. This result appears to indicate some amino acid sequence incompatibility in the chicken ICD with downstream factors or adaptors in the human cells. Deficits might also be caused by differences in intracellular trafficking or signaling potential, e. g. by interplay between heterologous ECD, TM and ICD. It is also possible that frequent non-synonymous SNPs of chicken TLR5 (in our case, two non-synonymous SNPs in the cloned chicken TLR5 ECD distant to LLR9) might be involved in modulating the TLR5 signaling capacities, which might explain discrepancies to previous studies^26,28^. Another factor determining TLR5 functionality is the transmembrane protein UNC93B1 which interacts with TLR5 close to its TM domain and influences TLR5 trafficking and localization^61,62^. We are currently investigating whether incompatibility with heterologous UNC93B1 might influence the functionality of various vertebrate TLR5; however, results have not been conclusive, since the expression of UNC93B1 after transient transfection mediated a strong reduction of TLR5 amounts in human cells (own unpublished results). Clearly, further analyses will be required to elucidate many of the remaining aspects of TLR5 protein-protein interactions, within and across species.

In our study, the responses to a reference flagellin, recombinant *Salmonella* FliC, confirm and expand the previously reported^5,27^ existence of host-species specific disparities between various tested vertebrate TLR5s at the level of NF-κB signaling. The significant concentration-dependent differences observed between human TLR5 and the chimeric receptors with regard to NF-κB activation might reflect amino acid differences within the TLR5 ECD, which may be responsible for altering direct interactions with the TLR5 ligand flagellin. In particular the amino acids 265 (chicken: T) and 275 (chicken and cattle: L) in LLR9, which were proposed to interact directly with flagellins of different bacterial species^48^, deviate in those two species from the TLR5 sequence of the other tested animal species. Despite this amino acid difference in LLR9, bovine TLR5 was fully functional. We cannot exclude that other sequence variation or variation in glycosylation between species in the ECDs outside of LLR9 impact on TLR5 structure, functionality and signal perception strength. Differences of TLR5 signal output at the species level were less pronounced with regard to IL-8 secretion, which may be partially due to the temporal accumulation of IL-8. This underscores the importance of a multi-pronged approach to comprehensively analyze cellular signaling pathways downstream of TLRs.

Reports of host species-specific TLR5 responses to flagellins have been published before^5,26-28^. Previously published comparative activation analysis of full length human, chicken and murine TLR5, carried out in heterologous expression systems, including hamster or human cellular background, showed distinctive recognition abilities in dose-response assays using purified bacterial flagellins, with overall higher responses by murine and chicken receptors compared to human TLR5 for most flagellins^5,28^. Moreover, mutations of single residues within murine TLR5 itself have implicated certain amino acids in distinctive species-specific recognition^5,28^. Previously reported host species-specific differences in response to purified flagellin were reproducible between mouse and human TLR5 (more robust activation by mTLR5^5,27,28^. Our present results for mTLR5 and hTLR5 dose-dependent activation support a similar conclusion. The divergent amino acid 268 between mouse (P) and human TLR5 (A), localized within the LLR9, was proposed to be responsible for the observed species-specific signaling differences^5^. Interestingly, this amino acid is also not conserved within the other tested vertebrate species (S for chicken, porcine and bovine TLR5). A prior study reported a species-specific difference comparing human and chicken TLR5, with a stronger response by chTLR5^28^. We note that the previous study^28^ relied on one selective reporter system in stably transfected cells, and the observed host species-specific differences in response to purified flagellins were small. Further aspects complicating the comparisons with results from different studies are the use of expression constructs which replace the natural signal peptide of the receptor with an artificial signal peptide^28^ which can affect trafficking and localization. Furthermore, the high abundance of SNPs within the coding sequence of TLR5, especially of domesticated animal breeds, may affect receptor signaling capacity. For instance, the prior study noted above^28^ utilized a chicken TLR5 variant harboring seven polymorphisms within the extracellular domain of TLR5, largely at positions highly conserved between vertebrate species, which might explain the observed discrepancies with our present results. Answering questions regarding the affinities of physical binding of specific flagellins to specific TLR5 ECDs requires complex biochemical approaches using purified TLR5 domains which have not been achieved yet. The functional implications of abundant SNPs in chicken TLR5 and differences between signaling results reported by various laboratories will require more detailed clarifications, for which we have now provided some basic tools.

The chimeric TLR5 ectodomain constructs described in this study were expressed and functional in human cells and were used for all further experiments to minimize ICD-dependent disparities between host-species. Previous reports regarding serovar-specific activation by *Salmonella* flagellins included only two serovars, *S*. Enteritidis^28^ and *S*. Typhimurium^5,28^, which did not allow conclusions. In our settings, purified flagellins of various *Salmonella* serovars did not show strong strain- or host-species specific differences in activation potential. This result may indicate that TLR5 ECDs do not provide a large discriminatory potential for flagellins of different *Salmonella* serovars. This data will still need to be expanded by more detailed dose-dependent activation studies. Supportive of this hypothesis, sequencing of flagellin genes of the tested *Salmonella* isolates revealed high sequence conservation within the TLR5-activating regions (e.g. amino acids Q89, N100, I411, L415), used for mutational approaches of bacterial flagellins in previous studies^5,27,28^. Amino acid conservation at these sites was independent of flagellar phase status or bacterial serovar. Lower NF-ĸB activation found for human and chimeric TLR5 receptors by flagellar fractions from two *S*. Enteritidis strains require further investigation. In the future, the chimeric test system can be used to characterize the flagellin-dependent responses in more detail, including flagellins from other bacterial species.

In contrast to the homogeneous effects of purified *Salmonella* flagellins, TLR5-dependent cell activation with the corresponding bacterial lysates revealed striking differences. The *Salmonella* lysates provoked differential activation outcomes, depending on the respective TLR5 variant expressed. Our present results permit us to conclude that specific inhibitory mechanisms acting on TLR5 signaling must exist in certain serovars, which may down-modulate the cell-activating responses induced by flagellins in a host species-specific manner. *Salmonella* serovars may be able to modulate TLR5 signaling to different extents, by flagellin-independent mechanisms. Modulation and inhibition of TLR signaling according to our present results seem to be specific for the TLR5 origin as well as *Salmonella* serovar-specific. Signal modulation could be dependent on bacterial outer membrane determinants or effectors of the virulence-associated Type III secretion systems, which might be accessible to enter the cells from bacterial lysates. Effectors such as TlpA (TIR-like protein A) or AvrA^63,64^ might mediate serovar-specific inhibition on TLRs, other TIR-domain containing adaptor proteins, or on NF-κB activation, respectively. This attractive hypothesis should be tested in the future.

Extending previous studies using only five different *C. jejuni* strains comparing activation of human and chicken TLR5^11,26,39,40^, we examined 17 preselected diverse *C. jejuni* isolates, including strains isolated from human and animal origin. All 17 isolates provided very little activation potential in cells expressing the chimeric TLR5 constructs. This suggests that *C. jejuni*, independent of host species, does not activate cells to a large extent via TLR5 ectodomains of any of the tested host species. This corroborates previous results obtained in human reporter cells, which showed that *C. jejuni* evades TLR5 recognition, and, via different molecules including glycans^65,66^ or the TLR5 ligand protein FlaC^38^, downmodulates TLR signaling. In line with these findings, purified flagellins of various selected *C. jejuni* isolates did not show ECD-dependent TLR5 activation of the tested vertebrate species, which confirms and expands previous studies^11,26,39,40^.

In conclusion, we have characterized signaling limitations for the expression of TLR5 variants from heterologous species in human cells and established a test system for the functional characterization of ligands and signal transduction by host-specific TLR5 ectodomains. *Salmonella* serovars appear to inhibit TLR5 signaling in a strain-specific manner by flagellin-independent mechanisms, while *C. jejuni* variants or flagellins did not activate any tested TLR5. The established test system will serve in the future to characterize TLR5 signaling requirements, activation and inhibitory effects by diverse ligands and bacteria in more detail.

## Materials and Methods

### Bacterial strains and culture conditions

Cloning was performed in *Escherichia coli* strains DH5α, MC1061, XL1-Blue (NEB) NEB5α (NEB). For overexpression of proteins, *E. coli* strain BL21(DE3) was used. *E. coli* was cultured in Luria–Bertani (LB) broth (Difco™ LB Agar, Lennox, BD Biosciences, Heidelberg, Germany) or on LB plates containing 1.5% Bacto agar. When appropriate, ampicillin (500 mg/l) was added to the medium. For the differential activation of cells, lysates and whole bacteria of various *S. enterica* serovars (24 strains in total) and 17 *C. jejuni* strains (Table S1) were used. Bacterial strains were collected and characterized by molecular typing in-house at Hannover Medical School. *C. jejuni* were cultured at 37 °C under microaerobic conditions (10% CO_2_, 5% O_2_, 85% N_2_) in vented jars on blood agar plates (Blood Agar Base II, Oxoid, Wesel, Germany), supplemented with 10% defibrinated horse blood (Oxoid) and standard antibiotics (10 mg/l vancomycin, 3.2 mg/l polymyxin B, 5 mg/l trimethoprim, 4 mg/l amphotericin B), or in brain-heart infusion broth (Oxoid) with the addition of 2.5 g/l yeast extract (Merck, Darmstadt, Germany). *Salmonella enterica* were grown on Columbia agar plates, containing 5% sheep blood at 37 °C. For bacterial lysate preparations, bacteria were grown on plates for approximately 24 h before harvest.

### Cell types and culture conditions

HEK293-T, HeLa or HEK-Blue Null1 cells (#hkb-null1, Invivogen) were used for transfection and coincubation assays and propagated in Dulbecco’s MEM (Biochrom, Berlin, Germany) supplemented with 10% [v/v] fetal bovine serum (FBS; Promocell). Growth medium of HEK-Blue Null1 cells was additionally supplemented with 100 µg/ml of zeocin as selective antibiotic. HD-11 chicken macrophage-like cell line used for nucleofection experiments was maintained in Iscove’s basal medium (IBM) supplemented with 10% [v/v] FBS. HD-11 cells stably transfected with the firefly luciferase gene under control of a NF-κB promoter were cultured in medium containing puromycin as selective antibiotic (5 g/liter). All cell lines were routinely kept at 37 °C in a 5% CO_2_ humidified atmosphere.

### DNA methods and cloning of TLR5 expression plasmids

(Schematic shown in Fig. S4) DNA methods were performed according to standard protocols using enzymes purchased from New England Biolabs (NEB, Ipswich, NJ, USA), Invitrogen (Carlsbad, California, USA) or Roche (Basel, Switzerland). Taq polymerase (Roche), Phusion polymerase (NEB) or Q5 polymerase (NEB) were used for PCRs. *TLR5* genes from different species (human, mouse, chicken, pig and cattle) were PCR- amplified using primers listed in Table S3. Specifically: amplification of the entire mouse *TLR5* was achieved by mTLR5_F2 and mTLR5_R2 primers and J774 cDNA (*Mus musculus*; balb/c mouse) as template. Complete chicken *TLR5* was amplified by chTLR5_F1 and chTLR5_R1 primers and HD-11 cDNA as template (chicken [*Gallus gallus*] breed: Polish Bantam), and complete pig *TLR5* by piTLR5_F1 and piTLR5_R1 primers and template cDNA from IPEC-J2 cells (porcine [*Sus scrofa*] breed: German Landrace). All of the amplified *TLR5* genes were cloned into pEF6-V5 expression vector (Novagen modified from^14^) using appropriate restriction enzymes. Bovine *TLR5* was recloned from commercially available pUNO1-bTLR05 plasmid (Invivogen, # puno1-btlr5; bovine [*Bos taurus*] breed: Holstein) into pEF6-V5 vector using boTLR5-BstXI and boTLR5_NotI_R2 primers. Sanger sequencing technology was applied for precise sequencing of the complete cloned plasmids to verify sequence accuracy (used primers depicted in Table S4; complete alignment of amino acid sequences of all full-length TLR5 clone inserts in Fig. S11). Additionally, chimeric TLR5 receptors containing extracellular domains of animal origin, linked to a human intracellular domain were designed and generated. Gene sequence coding for extracellular domains of TLR5 receptors from different animal species were PCR amplified using the following primers (see also Table S3): mouse: mTLR5_F2 mTLR5_R4; chicken: chTLR5_F1 and chTLR5_R2; pig: piTLR5_F2 and piTLR5_R2 and cattle: boTLR5_BstXI_F and boTLR5_BsaI_R. These amplified and correspondingly digested gene fragments were ligated to gene sequence coding for the intracellular domain of human TLR5 using an integrated *AflII* restriction site. The connection point of ECD and ICD domains was placed within the transmembrane part of the receptor (see Figs 1; S4) and leads to very few, mostly conservative amino acid exchanges (ch/h: none; pi/h: none; m/h: aa 651 Arg → Thr, aa 652 Ser → Gly, aa 653 Leu → Thr; bo/h: aa 636 Glu → Ser, aa 637 Ser → Gly, aa 638 Leu → Thr). The expression of all cloned receptors was controlled by Western Immunoblotting and Immunofluorescence (see Fig. 1). Exchanges of single nucleotides within plasmids, leading to site-directed amino acid exchanges, were generated by usage of Quick Change Site-Directed Mutagenesis Kit (Agilent) or Q5 kit (NEB) according to the manufacturers’ instructions (used primers listed in Table S5).

### Transient cell transfection or nucleofection with plasmid DNA and protein expression analysis

HEK293-T or HeLa cells were seeded in 24-well plates and HEK-Blue Null1 cells in 96-well plates. Cells were used for transfection experiments at 60% confluency. 30 min before transfection, the medium was changed to 0.5 ml (24-well plate) or 50 µl (96-well plate) OptiMEM medium (Gibco) containing 5% fetal calf serum (FCS). Indicated amounts (usually 200 to 50 ng) of plasmids of interest (Table S2 for plasmids used in this study), which were prepared by endo-free plasmid Midi-Kit (Qiagen, Hilden, Germany), were transfected using Lipofectamine 2000^TM^ (Invitrogen) according to the manufacturer’s instructions. Nucleofection of HD-11 cells was performed according to manufacturer’s instructions as for murine RAW264.7 cells using the SF cell line 4D-Nucleofector Kit (Lonza), and 4D-Nucleofector Device (Lonza) and 400 ng per well (24-well plate) or 2 µg per well applied DNA (6-well plate). Further incubation for 24 h or 48 h allowed the expression of transfected or nucleofected constructs (as indicated in main text and figure legends). Cell lysis and harvesting was performed directly within the wells on ice using modified radio-immunoprecipitation assay (RIPA) buffer (50 mM Tris-HCl, pH = 7.5, 150 mM NaCl, 1% Triton X-100, 1 mM EDTA, 1 mM EGTA, protease inhibitor cocktail Complete (Roche), phosphatase inhibitor cocktail PhosStop (Roche)). Analyses by Western immunoblotting were carried out for all human cell lines and HD-11 chicken cells to verify the protein expression levels of all constructs. For HD-11 cells nucleofected with plasmids, *tlr5* transcripts were also tested using cDNA and qPCR. The transcript amounts of the three transfected *tlr5* variants *htlr5*, *chtlr5*, *ch/htrlr5* were found to be comparable in this setting by qPCR. Intrinsic *tlr5* was expressed in HD-11 cells, but at about 17-fold lower amounts (qPCR) than *chtlr5* transcript expressed from a transfected chTLR5 plasmid. Intrinsic TLR5 led to about 2.5-fold increase in chIL-8 transcript (Fig. 5B), while transfected chTLR5 plasmid led to an about 6-fold increase in the same setting.

### Protein methods

Protein amounts were determined by bicinchoninic acid (BCA) Protein Assay (Thermo Scientific - Pierce, Rockford IL, USA) and protein analysis was achieved by separation on denaturing 12% sodium dodecyl sulphate (SDS) polyacrylamide gels and Western immunoblot detection according to standard methods^67^. Equal amounts (usually 30 µg) of protein were loaded on each gel lane. Antibodies for labeling are indicated in the corresponding results and listed in Table S6. Immuno-reactive bands were visualized by Super Signal West Pico chemiluminescent substrate (Pierce, Thermo Scientific, Bonn, Germany) and ECL hyperfilm (GE Healthcare, Piscataway NJ, USA). Additional normalization of gel loading was performed against the corresponding loading controls visualized by using anti-actin antibody (Chemicon, MAB1501; mouse monoclonal antibody; used at a 1:30,000 dilution). In the case of sequential antibody application, membranes were stripped with Restore™ Western Blot Stripping Buffer (Thermo Scientific – Pierce).

### Recombinant expression and purification of *S. enterica* serovar Typhimurium FliC

Expression and purification of *S. enterica* serovar Typhimurium FliC was performed according to our own published work^14,38^. For coincubation experiments of eukaryotic cells with purified flagellin, FliC was additionally purified by elution of the protein from an SDS gel in an Electro Eluter (Model 422, Bio-Rad, Munich, Germany). Eluted protein was dialysed several times against cell culture grade PBS using dialysis cassettes (Slide-A-Lyzer, MWCO 3.500 cut-off, Thermo Scientific). Purity and amounts of ultrapure recombinant flagellin were checked on Coomassie blue-stained SDS gels (see Fig. S1). Limulus assays (LAL chromogenic endpoint assay; Cambrex) of recombinant flagellins prepared by this method did not detect LPS above the detection limit of 1 endotoxin unit (EU) per μg of protein^14^, so that we do not expect contamination by other TLR ligands. Ultrapure flagellin was sonicated (1 min at 4 °C, power 5) in solution to expose the monomers, before all cell coincubations.

### Bacterial lysates and preparation of flagellar fractionations

Whole cell lysates were generated from bacteria grown for 1.5-2 d on blood agar plates and resuspended in NaCl (0.9%). Lysis was achieved via sonication (Branson Sonifier) 3 times for 3 min.

For flagellar fractions, one day-old bacteria (in post-exponential phase, when abundant extracellular flagella have been formed), grown on the corresponding blood agar plates, were resuspended in 0.9% NaCl at an O.D._600_ of 4 to final volume of 500 µl. Surface-associated proteins including flagella proteins were sheared off the bacteria by repeatedly (30 times) pushing the bacterial suspension through a 23-gauge needle. Separation from bacterial cells was achieved by differential centrifugation: 20 min at 9,000 × g, 4 °C, followed by ultracentrifugation for 1 h, 40,000 rpm, at 4 °C (Beckman Optima 100 ultracentrifuge). Surface-associated proteins were resuspended in Tris buffer (100 mM Tris-HCl, pH = 7,5). Enriched isolated flagellins were verified by Western immunoblotting using an anti-*E. coli*-flagellin antibody (Table S6; this antibody reacts strongly to *Salmonella* and *Campylobacter* flagellins and reveals exclusively the flagellin bands in Western immunoblots), and protein amounts were determined by comparison to a bovine serum albumin (BSA) standard on SDS PAGE.

### Coincubation of eukaryotic cells with recombinant flagellins, bacterial lysates or flagellin-enriched bacterial fractions

For coincubation experiments with purified flagellins or bacterial lysates, cells were seeded onto 96- (2 × 10^4^ cells in 100 µl per well), 24- (2 × 10^5^ cells in 1 ml per well) or 6-well plates (1 × 10^6^ cells in 3 ml per well) 24 h prior to the coincubation. Media were replaced 30 min before the coincubation starting. Flagellins and lysates were sonicated (1 min at 4 °C, power 5, in a Branson sonifier) shortly before the coincubations to release monomeric flagellin and disperse the suspensions well. Activation potential of purified flagellins or bacterial lysates on TLR5 receptors from different species was tested by coincubation of TLR5-transfected cells with various amounts of ultrapure and sonicated FliC (1 to 200 ng per well), bacterial lysates (100 ng per well) or flagellin-enriched bacterial surface-fractions (25 ng per well) for 4 h to 12 h. Optimal time frame and concentrations were determined by time- and concentration-dependent measurements (Fig. 3) and regression analysis (Fig. S6A). Activation of NF-κB was determined using HEK-Blue Null1 reporter cells in 96-well plate format. NF-κB-dependent production of secreted embryonic alkaline phosphatase (SEAP) was detected by application of HEK-Blue Detection Medium (Invivogen), which allows real-time detection of produced SEAP amounts by colorimetric measurements at 620 nm. NF-κB-dependent activation of single TLR5 receptors by bacterial lysates and bacterial flagellin-enriched fractions is always depicted relative to a respective reference, which is the corresponding TLR5-V5 construct activated by 20 ng of the reference flagellin (recombinant *Salmonella* FliC) and set to 100%. Background activation of empty vector-transfected and analogous lysate- or flagellin-activated cells was subtracted in order to obtain a TLR5 signaling specific read-out. We determined the expression of the TLR5 variants to be similar and comparable in HEK-Blue cells under the same transfection settings as in HEK293-T cells. For the analysis of NF-κB-mediated activation of chicken cells, stable luciferase HD-11 reporter cells were stimulated in a 96-well plate format and analyzed using SteadyGlo assay (Promega). HEK293-T or HeLa cells in 24-well plate format were used for transfection and subsequent coincubation experiments and for further quantitative analysis of cytokine secretion (IL-8). IL-8 in supernatants of activated cells was determined by a commercially available ELISA (IL-8 OptEIA^TM^ ELISA system, Becton Dickinson, Inc, BD Biosciences), according to the manufacturer’s recommendations. 6-well plate format was chosen for experiments with subsequent RNA preparations and RT-PCR analysis.

### RNA preparation and quantitative real-time (RT) PCR

RNA from nucleofected HD-11 cells was isolated as already described^38^. DNAse I treatment of the isolated RNA was performed with Turbo DNA-free Kit (Ambion) according to manufacturer’s instructions. 1 µg of total DNA-free RNA was used for cDNA synthesis with Superscript III reverse transcriptase (Invivogen) and oligo(dT) primers (Invivogen). 1 µl of synthesized cDNA was used for quantitative RT-PCR based on SYBR green (Qiagen) chemistry. Normalisation of the results was carried out according to transcriptional expression of chicken glyceraldehyde-3-phosphate dehydrogenase (chGAPDH). All primers used for RT-PCRs are listed in Table S7. RT-PCR reactions were performed in a Bio-Rad thermocycler (Bio-Rad C1000/CFX96 combined system) with following cycling conditions: denaturation for 10 min at 95 °C, amplification for 40 cycles at 95 °C for 15–45 s, 15 s at an annealing temperature optimized individually for each primer pair, 30 s at 72 °C. The expression of TLR5 variants after transfection was also probed at the cDNA level using qPCRs. It confirmed comparable *tlr5* transcript amounts of the hTLR5-, chTLR5- and ch/hTLR5-transfected HD-11 cells, while the non-transfected HD-11 cells had about 16-fold lower amounts of intrinsic *tlr5* transcript.

### Immunofluorescence-based automated quantification by Cytation 3

For expression-analysis of different TLR5 constructs, HEK293-T cells were seeded onto coverslips coated with 0.3% gelatin and transfected with TLR5-coding plasmids using Lipofectamine 2000^TM^. Two days later, cells were fixed with 2% paraformaldehyde in potassium phosphate buffer (pH = 7) two times for one h, followed by a quenching step with quenching buffer (0.1% glycine in PBS) over night at 4 °C. Afterwards, cells were washed three times with PBS, then blocked and simultaneously permeabilized for 30 min in buffer containing 1% BSA, 1% goat serum, 0.05% saponine, in PBS. Anti-V5 (1:500) primary antibody was applied over night at 4 °C in PBS containing 1% BSA and 1% FCS. After washing the cells three times using washing buffer (PBS with 0.1% BSA), secondary antibody goat anti-mouse Alexa Fluor^TM^ 488 (1:5,000) was applied in PBS with 1% BSA and 1% FCS for 30 min at room temperature. Cells were repeatedly washed again and finally counterstained for the nucleus with DAPI (1:5,000, Sigma-Aldrich) for 15 min. The coverslips were mounted using Mowiol 4–88 (Merck) supplemented with 2.5% DABCO (Sigma-Aldrich) on microscope slides. Anti-V5 antibody staining was always negative on non-transfected or empty-vector transfected cells. The green fluorescence channel was compensated for autofluorescence using non-transfected cells. Automated fluorescence microscopy for cellular expression analysis was performed using Cytation 3 Cell Imaging Multi-Mode Reader (Biotek) using constant, compensated, settings for the green fluorescence detection channel for all constructs (LED intensity: 10; Integration time: 136; Camera gain: 15.6) and the following cellular identification parameters (minimal size: 18 µm; maximal size: 34 µm, intensity threshold in the green fluorescence channel: 3,000). Automatically recorded doublet cells were excluded manually. Cells were also semi-automatically evaluated for nuclei(DAPI)-positive (all cells) versus nuclei and Alexa488-positive (green) cells, to determine the transfection efficiencies for each TLR5 construct. The transfection efficiencies were determined to be as follows:

hTLR5 = 57.44%; chTLR5 = 55.5%; ch/hTLR5 = 73.5%; mTLR5 = 67.76%; m/hTLR5 = 67.74%; pTLR5 = 50.72%; p/hTLR5 = 54.46%; bTLR5 = 47.61%; b/hTLR5 = 66.12%. Full-length and chimeric receptors of the same species show transfection efficiencies in a similar range.

## Electronic supplementary material

Supplementary Information
