# Supplementary material for: Functional expression of TLR5 of different vertebrate species and diversification in intestinal pathogen recognition
Source: Sci Rep. 2018 Jul 26;8:11287. doi: 10.1038/s41598-018-29371-0 (PMC6062626; doi:10.1038/s41598-018-29371-0)
Supplement: Supplementary file 1 — Supplementary Information [file 41598_2018_29371_MOESM1_ESM.pdf]

Supplementary Information

Functional expression of TLR5 from different vertebrate species and diversification towards intestinal pathogen recognition

Eugenia Faber [1], Karsten Tedin [2], Yvonne Speidel [1], Melanie M. Brinkmann [3], Christine Josenhans [1,4*]

[1] Medizinische Hochschule Hannover, Institute for Medical Microbiology, Carl-Neuberg-Strasse 1, 30625 Hannover, Germany
[1] DZIF-German Center for Infection Research, Partner site Hannover-Braunschweig, Germany
[2] Institute of Microbiology and Epizootics, Free University Berlin, Germany
[3] Helmholtz Center for Infection Research, Braunschweig, Germany
[4] Max von Pettenkofer Institute, Ludwig Maximilians University Munich, Pettenkoferstrasse 9a, 80336 Munich, Germany

*Address correspondence to:
Christine Josenhans; Josenhans@mvp.uni-muenchen.de

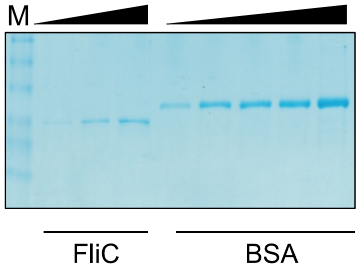

Faber *et al*., Figure S1

Fig. S1: determination of protein amount and purity of highly purified recombinant *S.* Typhimurium FliC. Different amounts of recombinantly expressed and highly purified *S.* Typhimurium FliC (used as reference protein for TLR5 activation) were loaded on an SDS gel and stained with Simply Blue Safestain (Novex): M: protein molecular mass standard (Bench Mark Prestained, Novex), lane 1 from left: 20 ng FliC; lane 2: 100 ng FliC, lane 3: 200 ng FliC, lane 4: 100 ng BSA, lane 5: 200 ng BSA, lane 6: 300 ng BSA, lane 7: 500 ng BSA, lane 8: 1 µg BSA.

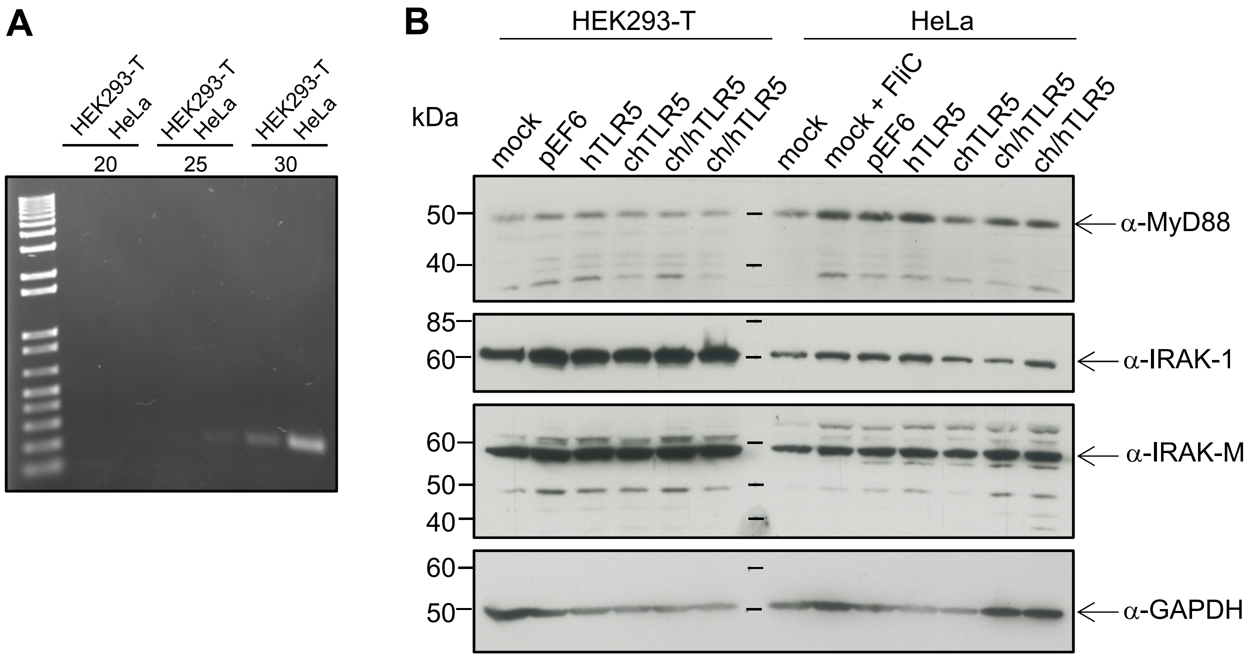

Faber *et al.*, Figure S2

Fig. S2: comparative expression of TLR signaling pathway components in HEK293-T and HeLa cells. A) Semiquantitative PCRs for MyD88 transcript were performed using cDNA of HEK293-T or HeLa cells as template and the following primers: hMyD88_F1 and hMyD88_R1. 5 µl of the amplified PCR product (after 20, 25 or 30 amplification cycles) was loaded on a 1% agarose gel. **B)** Western blot analysis using cleared lysates of empty vector (200 ng) or TLR5-V5 construct-transfected HEK293-T (h: 75 ng, ch: 200 ng, ch/h: 200 ng and 250 ng) or HeLa cells (h: 50 ng, ch: 500 ng, ch/h: 50 ng and 100 ng) using anti-MyD88, anti-IRAK-1, anti-IRAK-M and anti-GAPDH antibodies (see Table S6).

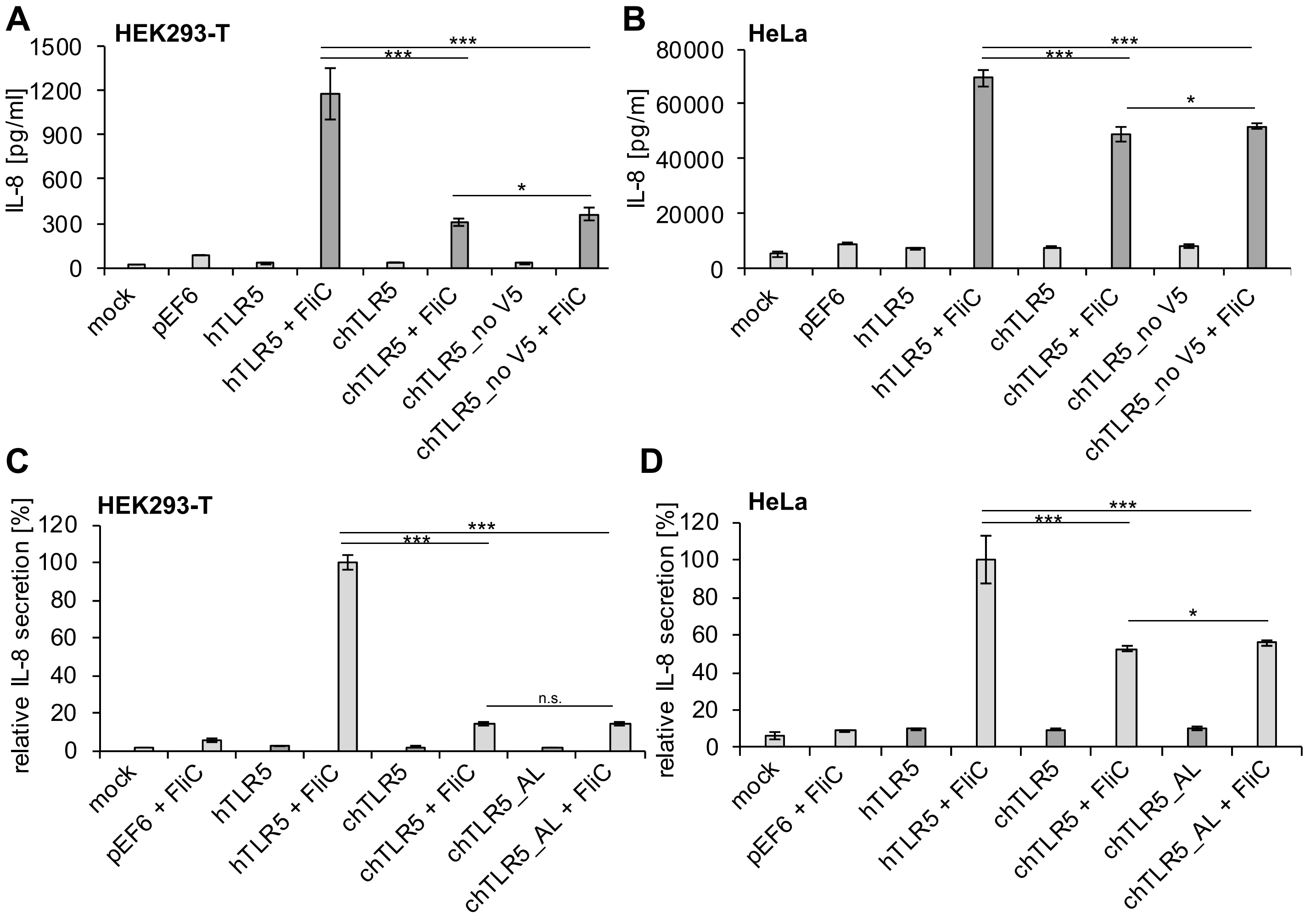

Faber *et al.*, Figure S3

Fig. S3: C-terminal V5-tag or an alternative 3' upstream sequence have minor influence on the activation efficiency of expression constructs for chicken TLR5 by *Salmonella* FliC. HEK293-T (**A, C**) or HeLa cells (**B, D**) were transiently transfected with empty vector pEF6-V5: 200 ng; human TLR5-V5: 100 ng; chicken TLR5-V5 or same construct without V5 tag: 200 ng. For comparison, expression constructs for chicken TLR5-V5 with alternative upstream (AL) sequences were analyzed. 48 h post transfection, cells were coincubated with purified recombinant *Salmonella* FliC (50 ng/well), followed by a measurement of secreted IL-8 in cell supernatants. **C** and **D**: IL-8 secretion of hTLR5-transfected and FliC-activated cells was set to 100 % (reference); relative IL-8 secretion of other constructs with regard to the reference is depicted in [%]. Mean and standard deviation from technical triplicates of biological duplicates of a representative experiment are shown. Significant differences between FliC-stimulated hTLR5 and activated chicken TLR5 variants as well as between activated chTLR5 and activated mutated chTLR5 variants are indicated by asterisks (Student's *t*-test, unpaired, two-tailed) as follows: *, $0.01<p<0.05$; **, $0.001<p<0.01$; and ***, $p<0.001$, n.s. non-significant.

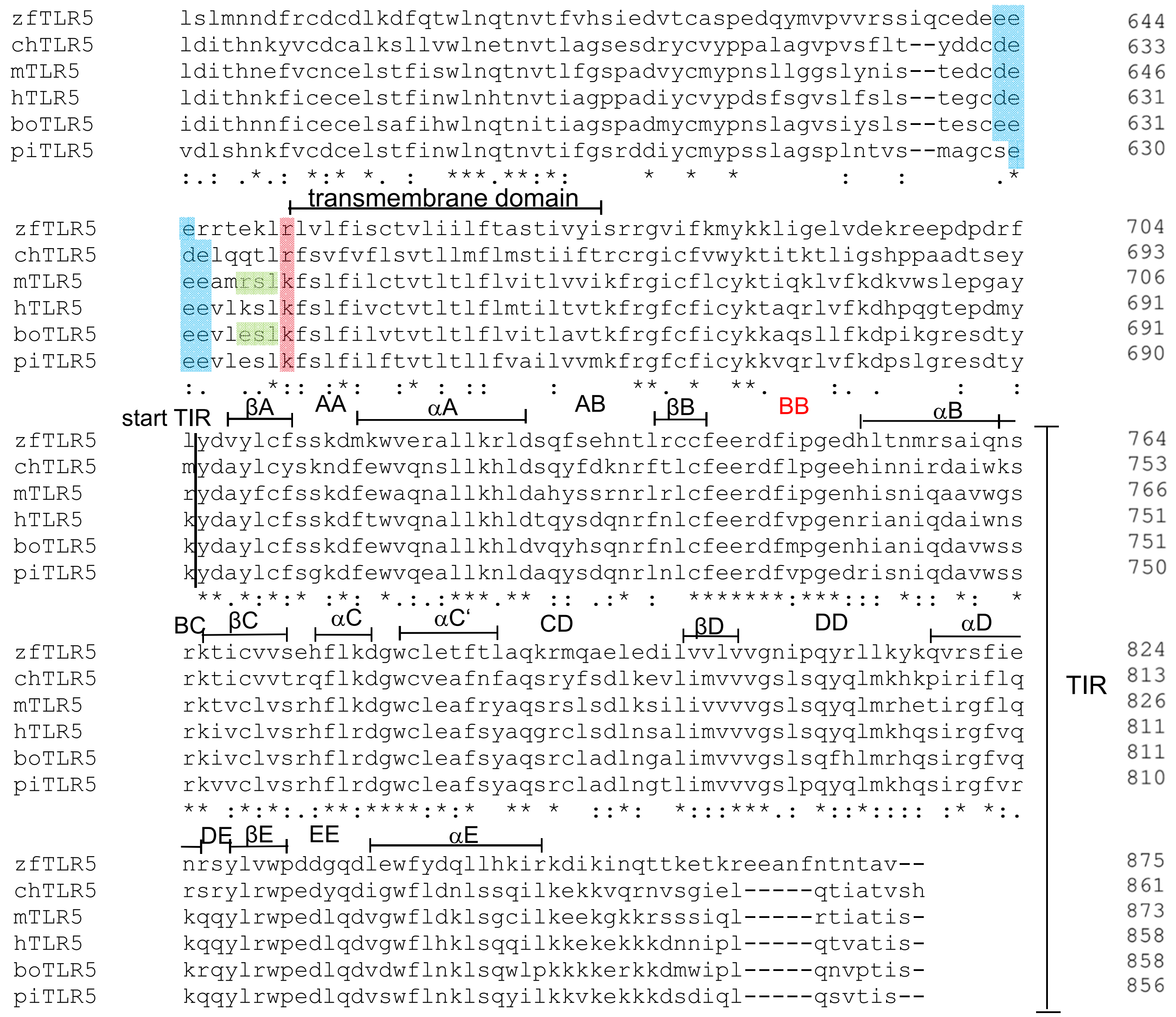

Faber *et al.*, Figure S4

Fig. S4: alignment of partial TLR5 amino acid sequences (focus and transmembrane and intracellular domains) from different vertebrate species. Amino acid sequences of TLR5 receptors from different vertebrate species (zf: zebrafish, ch: chicken, m: murine, h: human, bo: bovine, pi: porcine) were aligned using ClustalW2 (Larkin *et al.*, 2007). Structural components of intracellular TIR domains were assigned according to (Xu *et al.* 2000) and are depicted as abbreviations above the alignment. These components include the BB loop (BB in red) which is involved in TIR-TIR interactions. The start residue of the C-terminal human segment after the non-human N-terminal ECD domain within the chimeric receptor constructs is marked in red. Amino acid exchanges in chimeric mouse/human and bovine/human TLR5 generated in this study by the cloning strategy are marked in green and are as follows: m/h: RSL to TGT and bo/h: ESL to SGT. Acidic residues in the linker domain upstream of the transmembrane domain, which are most likely important for the interaction of TLR5 with UNC93B1 (as shown for mouse UNC93B1 and murine nucleotide-sensing TLRs (Kim *et al.*, 2013) and for proper TLR5 functionality (Huh *et al.*, 2013)), are not affected by the cloning strategy of the chimeric proteins and are shaded in blue.

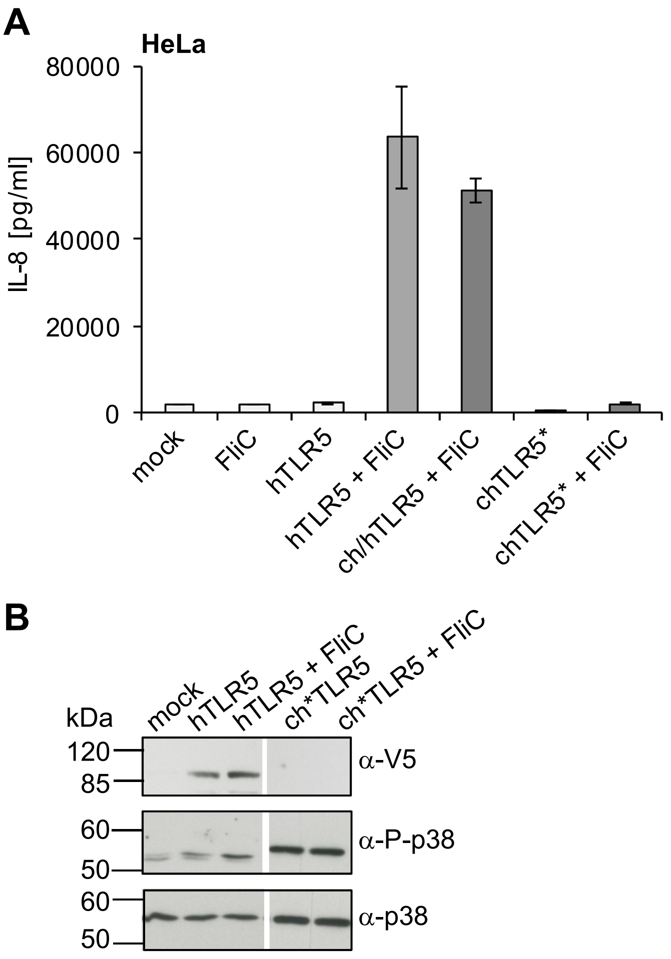

Faber *et al.*, Figure S5

Fig. S5: mutation of amino acid 744 in the chicken TIR domain to human counterpart results in abolished expression of receptor. HeLa cells were transiently transfected with human (h) TLR5-V5: 100 ng; chicken/human chimeric (ch/h) TLR5-V5: 100 ng or mutated chicken (ch*) TLR5-V5 (amino acid 744 N to A): 200 ng. 48 h post transfection, cells were coincubated with purified recombinant *Salmonella* FliC (50 ng/well), followed by a measurement of secreted IL-8 in cell supernatants. Mean and standard deviation from technical triplicates of biological duplicates of one representative experiment are shown. (**A**). Western Blot analysis of cleared lysates of transfected HeLa cells detected using anti-V5 antibody, anti-P-p38 antibody and anti-p38 antibody (**B**) (see Table S6).

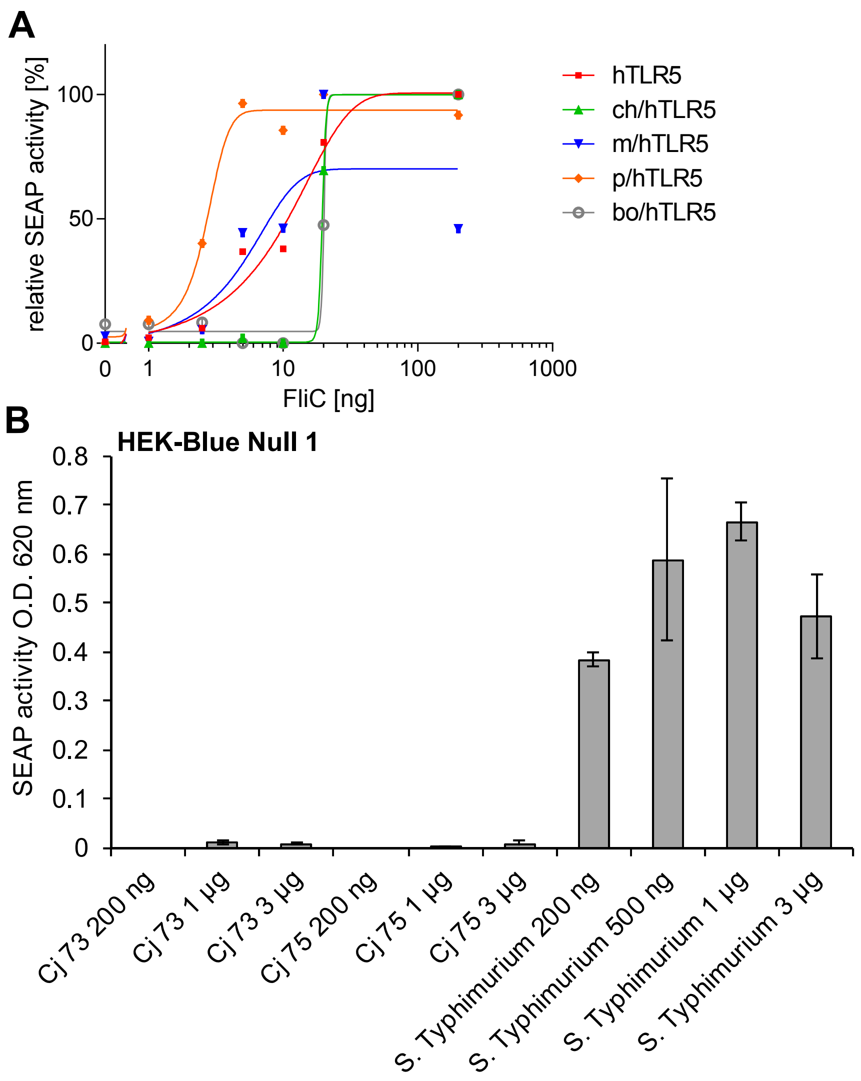

Faber *et al.*, Figure S6

Fig. S6: controls for experimental setup of ECD-dependent TLR5 signaling screening system. A) Non-linear regression curves of dose responses of chimeric TLR5 receptors for determination of optimal activation doses for the system (measured original values shown in Fig. 3 B). Values are depicted relative to a reference (corresponding TLR5 construct activated by maximal dosage of FliC), set as 100%. Regression was performed using GraphPad Prism 6 using the variable slope model (four parameter dose-response curve). **B)** NF-κB activation of human TLR5 by increasing amounts of *C. jejuni* and *S*. Typhimurium lysates (dose dependency determination). HEK-Blue Null1 NF-κB reporter cells were transiently transfected with 100 ng DNA of human TLR5-V5. After 24 hours, transfected cells were coincubated with 200 ng to 3 µg protein content of *C. jejuni* (isolate Cj 73 or Cj 75) or *S. Typhimurium* whole bacterial lysates per well for 11 h; NF-κB-dependent SEAP production was determined by colorimetric measurements at 620 nm (see Methods). Absolute values of the colorimetric tests are shown. Background activation was determined by activation of empty vector-transfected cells by the corresponding lysate and subtracted from each test value to quantify specifically the TLR5-dependent activation. Mean and standard deviation from technical triplicates of a representative experiment are shown.

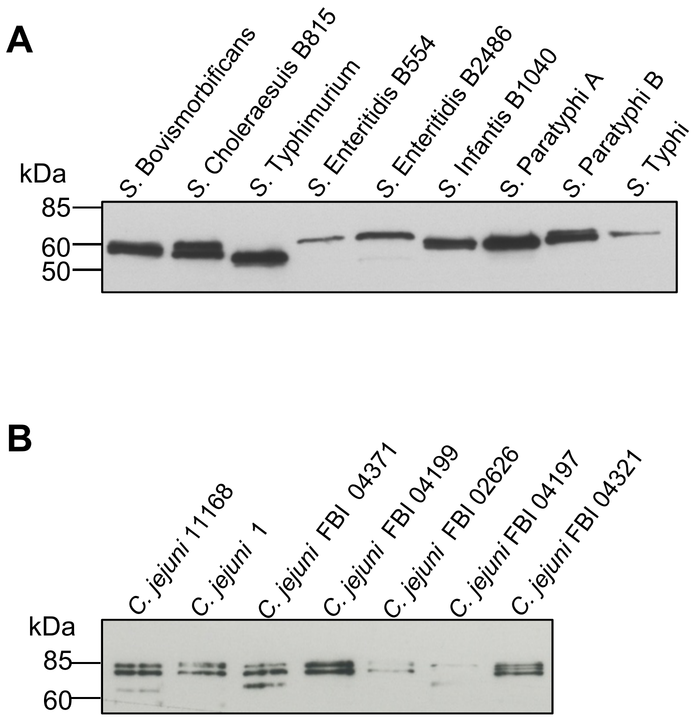

Faber *et al.*, Figure S7

Fig. S7: comparison of flagellin expression by selected *Salmonella enterica* serovars (A) and *C. jejuni* (B) isolates in whole bacterial lysates. Western Blot analysis of selected *Salmonella* or *C. jejuni* lysates (5 µg total protein/lane) using anti-*E. coli* flagellin antibody, which specifically recognizes flagellin proteins in *Salmonella* and *Campylobacter*. *S.* Enteritidis B554 only expresses FliC, while *S.* Choleraesuis B815 expresses only FljB.

A ECD

	human	chicken	mouse	pig	cattle
human		49.1	70.3	77.0	78.4
chicken	49.1		46.5	50.2	49.3
mouse	70.3	46.5		68.0	67.1
pig	77.0	50.2	68.0		79.8
cattle	78.4	49.3	67.1	79.8	

B TM + TIR

	human	chicken	mouse	pig	cattle
human		59.7	74.1	80.9	81.4
chicken	59.7		59.7	52.9	55.2
mouse	74.1	59.7		71.4	70.9
pig	80.9	52.9	71.4		80.9
cattle	81.4	55.2	70.9	80.9	

C complete

	human	chicken	mouse	pig	cattle
human		51.9	71.2	78.0	78.7
chicken	51.9		49.8	50.9	50.8
mouse	71.2	49.8		68.8	68.0
pig	78.0	50.9	68.8		80.1
cattle	78.7	50.8	68.0	80.1	

D ECD

	human	chicken	mouse	pig	cattle
human		66.6	79.6	86.5	87.1
chicken	66.6		63.9	66.1	66.3
mouse	79.6	63.9		77.8	77.8
pig	86.5	66.1	77.8		88.6
cattle	87.1	66.3	77.8	88.6	

E TM + TIR

	human	chicken	mouse	pig	cattle
human		76.5	83.6	87.3	88.2
chicken	76.5		77.4	74.7	73.3
mouse	83.6	77.4		81.8	81.4
pig	87.3	74.7	81.8		87.3
cattle	88.2	77.3	81.4	87.3	

F complete

	human	chicken	mouse	pig	cattle
human		69.1	80.6	86.7	86.9
chicken	69.1		67.3	68.3	68.1
mouse	80.6	67.3		78.8	78.7
pig	86.7	68.3	78.8		88.2
cattle	86.9	68.1	78.7	88.2	

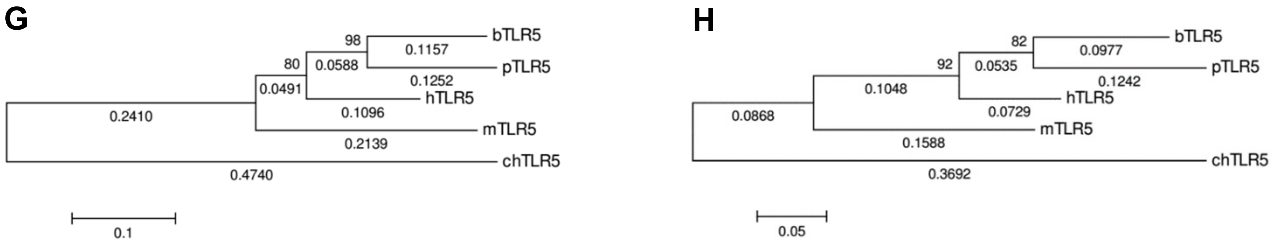

Faber *et al.*, Figure S8

Fig. S8: pairwise amino acid sequence comparison of TLR5 from various vertebrate species. A to **F:** matrix depiction of pairwise amino acid sequence comparison of TLR5 from different vertebrate species regarding amino acid sequence identity (in [%], **A** to **C**) or amino acid sequence similarity (in [%], **D** to **F**) using pairwise alignment and final analysis with Needle (http://www.ebi.ac.uk/Tools/psa/emboss_needle/). ECD: ectodomain, TM: transmembrane domain, TIR: Toll/interleukin-1 receptor homology domain. ECD (**A**, **D**), TM plus TIR (**B**, **E**) and complete sequences (**C**, **F**) were analyzed separately. **G** and **H**) Maximum Likelihood Trees of ECD (**G**) and TM plus TIR (**H**) of TLR5 from different species (human (h), chicken (ch), mouse (m), pig (p) and bovine (b) TLR5) generated with MEGA5.2 (Tamura *et al.*, 2011) including 500 bootstrap repetitions.

```
                        *        20         *        40         *
B554_FliC   : -LNKSQSSLSSAIERLSSGLRINSAKDDAAGQAIANRFTSNIKGLTQASRNANDGIS :  56
B2359_FliC  : -LNKSQSSLSSAIERLSSGLRINSAKDDAAGQAIANRFTSNIKGLTQASRNANDGIS :  56
B2486_FliC  : -LNKSQSSLSSAIERLSSGLRINSAKDDAAGQAIANRFTSNIKGLTQASRNANDGIS :  56
B2334_FliC  : -LNKSQSSLSSAIERLSSGLRINSAKDDAAGQAIANRFTSNIKGLTQASRNANDGIS :  56
R160_FliC   : -LNKSQSALGTAIERLSSGLRINSAKDDAAGQAIANRFTANIKGLTQASRNANDGIS :  56
B1533_FliC  : -LNKSQSALGTAIERLSSGLRINSAKDDAAGQAIANRFTANIKGLTQASRNANDGIS :  56
B1830_FliC  : -LNKSQSAXGTAIERXSXGLRINSAKDDAAGQAIANRXTANIKGLTQASRNANDGIS :  56
B1040_FliC  : -LNKSQSALGTAIERLSSGLRINSAKDDAAGQAIANRFTANIKGLTQASRNANDGIS :  56
B1085_FliC  : -LNKSQSALGTAIERLSSGLRINSAKDDAAGQAIANRFTANIKGLTQASRNANDGIS :  56
B2075_FliC  : -LNKSQSALGTAIERLSSGLRINSAKDDAAGQAIANRFTANIKGLTQASRNANDGIS :  56
B2078_FliC  : -LNKSQSALGTAIERLSSGLRINSAKDDAAGQAIANRFTANIKGLTQASRNANDGIS :  56
B2183_FliC  : -LNKSQSALGTAIERLSSGLRINSAKDDAAGQAIANRFTANIKGLTQASRNANDGIS :  56
B2257_FliC  : -LNKSQSALGTAIERLSSGLRINSAKDDAAGQAIANRFTANIKGLTQASRNANDGIS :  56
B2396_FliC  : -LNKSQSALGTAIERLSSGLRINSAKDDAAGQAIANRFTANIKGLTQASRNANDGIS :  56
xy82_FliC   : -LNKSQSALGTAIERLSSGLRINSAKDDAAGQAIANRFTANIKGLTQASRNANDGIS :  56
R269_FliC   : -LNKSQSALGTAIERLSSGLRINSAKDDAAGQAIANRFTANIKGLTQASRNANDGIS :  56
xy810_FliC  : -LNKSQSALGTAIERLSSGLRINSAKDDAAGQAIANRFTANIKGLTQASRNANDGIS :  56
B571_FliC   : ----PQSALGTAIERLSSGLRINSAKDDAAGQAIANRFTANIKGLTQASRNANDGIS :  53
B1951_FliC  : -LNKSQSALGTAIERLSSGLRINSAKDDAAGQAIANRFTANIKGLTQASRNANDGIS :  56
B560_FliC   : -LNKSQSALGTAIERLSSGLRINSAKDDAAGQAIANRFTANIKGLTQASRNANDGIS :  57
B558_FliC   : -LNKSQSALGTAIERLSSGLRINSAKDDAAGQAIANRFTANIKGLTQASRNANDGIS :  56
                   QS   3AIER S GLRINSAKDDAAGQAIANR T NIKGLTQASRNANDGIS

                60         *        80         *       100         *
B554_FliC   : IAQTTEGALNEINNNLQRVRELSVQATNGTNSDSDLKSIQDEIQQRLEEIDRVSNQT : 113
B2359_FliC  : IAQTTEGALNEINNNLQRVRELSVQATNGTNSDSDLKSIQDEIQQRLEEIDRVSNQT : 113
B2486_FliC  : IAQTTEGALNEINNNLQRVRELSVQATNGTNSDSDLKSIQDEIQQRLEEIDRVSNQT : 113
B2334_FliC  : IAQTTEGALNEINNNLQRVRELSVQATNGTNSDSDLKSIQDEIQQRLEEIDRVSNQT : 113
R160_FliC   : IAQTTEGALNEINNNLQRVRELAVQSANSTNSQSDLDSIQAEITQRLNEIDRVSGQT : 113
B1533_FliC  : IAQTTEGALNEINNNLQRVRELAVQSANGTNSQSDLDSIQAEITQRLNEIDRVSGQT : 113
B1830_FliC  : XAQTTEGALNEINNNLQRVRELAVQSANGTNSQSDLDSIQAEITQRLNEIDRVSGQT : 113
B1040_FliC  : IAQTTEGALNEINNNLQRVRELAVQSANSTNSQSDLDSIQAEITQRLNEIDRVSGQT : 113
B1085_FliC  : IAQTTEGALNEINNNLQRVRELAVQSANSTNSQSDLDSIQAEITQRLNEIDRVSGQT : 113
B2075_FliC  : IAQTTEGALNEINNNLQRVRELAVQSANSTNSQSDLDSIQAEITQRLNEIDRVSGQT : 113
B2078_FliC  : IAQTTEGALNEINNNLQRVRELAVQSANSTNSQSDLDSIQAEITQRLNEIDRVSGQT : 113
B2183_FliC  : IAQTTEGALNEINNNLQRVRELAVQSANSTNSQSDLDSIQAEITQRLNEIDRVSGQT : 113
B2257_FliC  : IAQTTEGALNEINNNLQRVRELAVQSANSTNSQSDLDSIQAEITQRLNEIDRVSGQT : 113
B2396_FliC  : IAQTTEGALNEINNNLQRVRELAVQSANSTNSQSDLDSIQAEITQRLNEIDRVSGQT : 113
xy82_FliC   : IAQTTEGALNEINNNLQRVRELAVQSANSTNSQSDLDSIQAEITQRLNEIDRVSGQT : 113
R269_FliC   : IAQTTEGALNEINNNLQRVRELAVQSANSTNSQSDLDSIQAEITQRLNEIDRVSGQT : 113
xy810_FliC  : IAQTTEGALNEINNNLQRVRELAVQSANSTNSQSDLDSIQAEITQRLNEIDRVSGQT : 113
B571_FliC   : IAQTTEGALNEINNNLQRVRELAVQSANSTNSQSDLDSIQAEITQRLNEIDRVSGQT : 110
B1951_FliC  : IAQTTEGALNEINNNLQRVRELAVQSANSTNSQSDLDSIQAEITQRLNEIDRVSGQT : 113
B560_FliC   : XAQTTEGALNEINNNLQRVRELAVQSANSTNSQSDLDSIQAEITQRLNEIDRVSGQT : 114
B558_FliC   : IAQTTEGALNEINNNLQRVRELAVQSANSTNSQSDLDSIQAEITQRLNEIDRVSGQT : 113
               AQTTEGALNEINNNLQRVREL VQ  N TNS SDL SIQ EI  QRL EIDRVS QT

                 120         *       140         *       160         *
B554_FliC   : QFNGVKVLSQDNQMKIQVGANDGETITIDLQKIDVKSLGLDGFNVNGPKEATVGDLK : 170
B2359_FliC  : QFNGVKVLSQDNQMKIQVGANDGETITIDLQKIDVKSLGLDGFNVNGPKEATVGDLK : 170
B2486_FliC  : QFNGVKVLSQDNQMKIQVGANDGETITIDLQKIDVKSLGLDGFNVNGPKEATVGDLK : 170
B2334_FliC  : QFNGVKVLSQDNQMKIQVGANDGETITIDLQKIDVKSLGLDGFNVNGPKEATVGDLK : 170
R160_FliC   : QFNGVKVLAQDNTLTIQVGANDGETIDIDLKQINSQTLGLDTLSVQDAYTPKGTAVT : 170
B1533_FliC  : QFNGVKVLAQDNTLTIQVGANDGETIDIDLKEISSKTLGLDKPNVQDAYTPKETAVT : 170
B1830_FliC  : QXNGVKVLAQDNTLTIQVGANDGETIDIDLKEISSKTLGLDKLNVQDAYTPKETAVT : 170
B1040_FliC  : QFNGVKVLAQDNTLTIQVGANDGETIDIDLKQINSQTLGLDTLNVQQKYKVSDTAAT : 170
B1085_FliC  : QFNGVKVLAQDNTLTIQVGANDGETIDIDLKQINSQTLGLDTLNVQQKYKVSDTAAT : 170
B2075_FliC  : QFNGVKVLAQDNTLTIQVGANDGETIDIDLKQINSQTLGLDTLNVQQKYKVSDTAAT : 170
B2078_FliC  : QFNGVKVLAQDNTLTIQVGANDGETIDIDLKQINSQTLGLDTLNVQQKYKVSDTAAT : 170
B2183_FliC  : QFNGVKVLAQDNTLTIQVGANDGETIDIDLKQINSQTLGLDTLNVQQKYKVSDTAAT : 170
B2257_FliC  : QFNGVKVLAQDNTLTIQVGANDGETIDIDLKQINSQTLGLDTLNVQQKYKVSDTAAT : 170
B2396_FliC  : QFNGVKVLAQDNTLTIQVGANDGETIDIDLKQINSQTLGLDTLNVQQKYKVSDTAAT : 170
xy82_FliC   : QFNGVKVLAQDNTLTIQVGANDGETIDIDLKQINSQTLGLDTLNVQQKYKVSDTAAT : 170
R269_FliC   : QFNGVKVLAQDNTLTIQVGANDGETIDIDLKQINSQTLGLDTLNVQQKYKVSDTAAT : 170
xy810_FliC  : QFNGVKVLAQDNTLTIQVGANDGETIDIDLKQINSQTLGLDTLNVQKKYDVSDTAVA : 170
B571_FliC   : QFNGVKVLAQDNTLTIQVGANDGETIDIDLKQINSQTLGLDTLNVQKAYDVQATVEN : 167
B1951_FliC  : QFNGVKVLAQDNTLTIQVGANDGETIDIDLKQINSQTLGLDTLNVQKKYDVKSEAVT : 170
B560_FliC   : QFNGVKVLAQDNTLTIQVGANDGETIDIDLKQINSQTLGLDTLNVQKKYDVDSTGVT : 171
B558_FliC   : QFNGVKVLAQDNTLTIQVGANDGETIDIDLKQINSQTLGLDTLNVQKKYDVDSTGVT : 170
              Q NGVKVL QDN 6 IQVGANDGETI IDL  I   3LGLD    V
```

Continued on next page

```
                             180         *        200         *        220
B554_FliC  : SSFKNVTGYDTYAAGADKYRVDINSGAVVTDAAAPDKVYVNAANGQLTTDDAENNTA : 227
B2359_FliC : SSFKNVTGYDTYAAGADKYRVDINSGAVVTDAAAPDKVYVNAANGQLTTDDAENNTA : 227
B2486_FliC : SSFKNVTGYDTYAAGADKYRVDINSGAVVTDAAAPDKVYVNAANGQLTTDDAENNTA : 227
B2334_FliC : SSFKNVTGYDTYAAGADKYRVDINSGAVVTDAVAPDKVYVNAANGQLTTDDAENNTA : 227
R160_FliC  : RDVTTYKNGGTTLTAPNA-------AAIDTALGTTGAA--GTAAVKFKDGNY----- : 213
B1533_FliC : VDKTTYKNGTDPITAQSN-------TDIQTAIGGGATG--VTGADIKFKDGQYYLD- : 217
B1830_FliC : VDKTTYKNGTDPITAQSN-------TDIQTAIGGGATG--VTGADIKFKDGQYYLD- : 217
B1040_FliC : VTGYTDSATAIDKSTFAA-------SATTLGGTPAITG--DLK-FDDTTGKYY---- : 213
B1085_FliC : VTGYTDSATAIDKSTFAA-------SATTLGGTPAITG--DLK-FDDTTGKYY---- : 213
B2075_FliC : VTGYTDSATAIDKSTFAA-------SATTLGGTPAITG--DLK-FDDTTGKYY---- : 213
B2078_FliC : VTGYTDSATAIDKSTFAA-------SATTLGGTPAITG--DLK-FDDTTGKYY---- : 213
B2183_FliC : VTGYTDSATAIDKSTFAA-------SATTLGGTPAITG--DLK-FDDTTGKYY---- : 213
B2257_FliC : VTGYTDSATAIDKSTFAA-------SATTLGGTPAITG--DLK-FDDTTGKYY---- : 213
B2396_FliC : VTGYTDSATAIDKSTFAA-------SATTLGGTPAITG--DLK-FDDTTGKYY---- : 213
xy82_FliC  : VTGYTDSATAIDKSTFAA-------SATTLGGTPAITG--DLK-FDDTTGKYY---- : 213
R269_FliC  : VTGYADTTIALDNSTFKA-------SATGLGGTDQKID--GDL-KFDDTTGKY---- : 213
xy810_FliC : ASYSDSKQNIA----VPD-------KTAITAKIGAATS--GGAGIKADISFKDGKY- : 213
B571_FliC  : SVKLD-TSALTADAIKGG-------VTGATTAGALK-D--GKVYSNGTDYYVEV--- : 210
B1951_FliC : PSATLSTTALDGAGLKT--------GTGSTTDTGSIKD--GKVYYNSTSKNYYVEV- : 216
B560_FliC  : QSLDLKTAGITGATLKAG-------ITGTTTETGSVKD--GKVYYDADSKNYYVEV- : 218
B558_FliC  : QSLDLKTAGITGATLKAG-------ITGTTTETGSVKD--GKVYYDADSKNYYVEV- : 217

               *        240         *        260         *        280
B554_FliC  : VDLFKTTKSTAGTAEAKAIAGAIKGGKEGDTFDYKGVTFTI-D--TKTGDDGNGKVS : 281
B2359_FliC : VDLFKTTKSTAGTAEAKAIAGAIKGGKEGDTFDYKGVTFTI-D--TKTGDDGNGKVS : 281
B2486_FliC : VDLFKTTKSTAGTAEAKAIAGAIKGGKEGDTFDYKGVTFTI-D--TKTGDDGNGKVS : 281
B2334_FliC : VDLFKTTKSTAGTAEAKAIAGAIKGGKEGDTFDYKGVTFTI-D--TKTGDDGNGKVS : 281
R160_FliC  : ------FVEVTGTT--------KDGLYEATVDAAGAVTMTANKATVTGASTVTENQI : 256
B1533_FliC : ----VKGGASAGVYKATYDET-TKKVNIDTTDKTPLATAEATAI-RGTATITHNQIA : 268
B1830_FliC : ----VKGGASAGVYKATYDET-TKKVNIDTTDKTPLATAEATAI-RGTATITHNQIA : 268
B1040_FliC : ----ADVS---GTTAKDG-------VYEVTVAADGK-VTLTGTP-TGPITAGFPSTA : 254
B1085_FliC : ----ADVS---GTTAKDG-------VYEVTVAADGK-VTLTGTP-TGPITAGFPSTA : 254
B2075_FliC : ----ADVS---GTTAKDG-------VYEVTVAADGK-VTLTGTP-TGPITAGFPSTA : 254
B2078_FliC : ----ADVS---GTTAKDG-------VYEVTVAADGK-VTLTGTP-TGPITAGFPSTA : 254
B2183_FliC : ----ADVS---GTTAKDG-------VYEVTVAADGK-VTLTGTP-TGPITAGFPSTA : 254
B2257_FliC : ----ADVS---GTTAKDG-------VYEVTVAADGK-VTLTGTP-TGPITAGFPSTA : 254
B2396_FliC : ----ADVS---GTTAKDG-------VYEVTVAADGK-VTLTGTP-TGPITAGFPSTA : 254
xy82_FliC  : ----ADVS---GTTAKDG-------VYEVTVAADGK-VTLTGTP-TGPITAGFPSTA : 254
R269_FliC  : ----YAKV---TVTGGTGK---DGYYEVSVDKTNGE-VTLAGGA-TSPLTGGLPATA : 258
xy810_FliC : ----YATVSGYDDAADTDK---NGTYEVTVAADTGAVTFATRPT-VVDLPTDAKAVS : 262
B571_FliC  : ----SFADATDS-----GK---NGFLKVDVNTTTGAVTVPAAAA-NTVAAKPAGVSE : 254
B1951_FliC : ----EFTDATDQ-----TN---KGGFY-KVNVADDGAVTMTAAT-TKEATTPTGITE : 259
B560_FliC  : ----DFTDTTDK-----AA---HAGFYKADVDADGNVSLATGAT-KEAKPTNAVEVE : 262
B558_FliC  : ----DFTDTTDK-----AA---HAGFYKADVDADGNVSLATGAT-KEAKPTNAVEVE : 261

                 *        300         *        320         *        340
B554_FliC  : TTINGEKVTLTVADIATGATDVNAATLQSSKNVYTSVVNGQFTFDDKTK-NESAKLS : 337
B2359_FliC : TTINGEKVTLTVADIATGATDVNAATLQSSKNVYTSVVNGQFTFDDKTK-NESAKLS : 337
B2486_FliC : TTINGEKVTLTVADIATGATDVNAATLQSSKNVYTSVVNGQFTFDDKTK-NESAKLS : 337
B2334_FliC : TTINGEKVTLTVADIAIGAADVNAATLQSSKNVYTSVVNGQFTFDDKTK-NESAKLS : 337
R160_FliC  : VDAVTPTPVD---TVAAATALTNAGVTGA-TGNTSLVKMSFEDKNGKVTDAGYALKV : 309
B1533_FliC : EVTKEGVDTTTVAAQLAAAGVTGAD-----KDNTSLVKLSFEDKNGKVIDGGYAVKM : 320
B1830_FliC : EVTKEGVDTTTVAAQLAAAGVTGAD-----KDNTSLVKLSFEDKNGKVIDGGYAVKM : 320
B1040_FliC : TKDVKQTQQENADLTEAKAALTAAGVAA--AGTASVVKMSYTDNNGKTIDGGLAV-K : 308
B1085_FliC : TKDVKQTQQENADLTEAKAALTAAGVAA--AGTASVVKMSYTDNNGKTIDGGLAV-K : 308
B2075_FliC : TKDVKQTQQENADLTEAKAALTAAGVAA--AGTASVVKMSYTDNNGKTIDGGLAV-K : 308
B2078_FliC : TKDVKQTQQENADLTEAKAALTAAGVAA--AGTASVVKMSYTDNNGKTIDGGLAV-K : 308
B2183_FliC : TKDVKQTQQENADLTEAKAALTAAGVAA--AGTASVVKMSYTDNNGKTIDGGLAV-K : 308
B2257_FliC : TKDVKQTQQENADLTEAKAALTAAGVAA--AGTASVVKMSYTDNNGKTIDGGLAV-K : 308
B2396_FliC : TKDVKQTQQENADLTEAKAALTAAGVAA--AGTASVVKMSYTDNNGKTIDGGLAV-K : 308
xy82_FliC  : TKDVKQTQQENADLTEAKAALTAAGVAA--AGTASVVKMSYTDNNGKTIDGGLAV-K : 308
R269_FliC  : TEDVKNVQVANADLTEAKAALTA--AGV--TGTASVVKMSYTDNNGKTIDGGLAV-K : 310
xy810_FliC : KVQQNDTEIA---ATNAKAALKAAGVADAEADTATLVKMSYTDNNGKVIDGGFAFKT : 316
B571_FliC  : VTEVQGLNTP---SSAVQDQLTAAGVSAADAAKSEVVKMSYTDKNGKTIDGGFGVKV : 308
B1951_FliC : VTQVQKPVAA---PA--AIQAQLTAAHVTGADTAEMVKMSYTDKNGKTIDGGFGVKV : 311
B560_FliC  : KTIDEKPLKA---SSSVQDALKASGIADAVAEAATVVKMSYTDKNGKTIDGGYGIKV : 316
B558_FliC  : KTIDEKPLKA---SSSVQDALKASGIADAVAEAATVVKMSYTDKNGKTIDGGYGIKV : 315
                                                   V      1 K
```

Continued on next page

```
                         *        360         *        380         *
B554_FliC   : DLEANN-AVKGESKITVNGAEYTANATGDKITL----------------AGKTMFID : 377
B2359_FliC  : DLEANN-AVKGESKITVNGAEYTANATGDKITL----------------AGKTMFID : 377
B2486_FliC  : DLEANN-AVKGESKITVNGAEYTANATGDKITL----------------AGKTMFID : 377
B2334_FliC  : DLEANN-AVKGESKITVNGAEYTANATGDKITL----------------AGKTMFID : 377
R160_FliC   : GNDYYAADYDEKTGEIKAKTVNYTDATGATKTGAVKFGGANGKTEVVTTVDGNTYQA : 366
B1533_FliC  : GDDFYAATYDEKTGAITAKTTTYTDGTGVAQTGAVKFGGANGKSEVVTATDGKTYLA : 377
B1830_FliC  : GDDFYAATYDEKTGAITAKTTTYTDGTGVAQTGAVKFGGANGKSEVVTATDGKTYLA : 377
B1040_FliC  : VGDDYYSATQNKDGSISINTTKYTADDGTSKTALNKLGGADGKTEVVSIGGKTYAAS : 365
B1085_FliC  : VGDDYYSATQNKDGSISINTTKYTADDGTSKTALNKLGGADGKTEVVSIGGKTYAAS : 365
B2075_FliC  : VGDDYYSATQNKDGSISINTTKYTADDGTSKTALNKLGGADGKTEVVSIGGKTYAAS : 365
B2078_FliC  : VGDDYYSATQNKDGSISINTTKYTADDGTSKTALNKLGGADGKTEVVSIGGKTYAAS : 365
B2183_FliC  : VGDDYYSATQNKDGSISINTTKYTADDGTSKTALNKLGGADGKTEVVSIGGKTYAAS : 365
B2257_FliC  : VGDDYYSATQNKDGSISINTTKYTADDGTSKTALNKLGGADGKTEVVSIGGKTYAAS : 365
B2396_FliC  : VGDDYYSATQNKDGSISINTTKYTADDGTSKTALNKLGGADGKTEVVSIGGKTYAAS : 365
xy82_FliC   : VGDDYYSATQNKDGSISINTTKYTADDGTSKTALNKLGGADGKTEVVSIGGKTYAAS : 365
R269_FliC   : VGDDYYSATQNKDGSISINTTKYTADDGTSKTALNKLGGADGKTEVVSIGGKTYAAS : 367
xy810_FliC  : SGGYYAASVDKSGAASLKVTSYVDATTGTEKTAANKLGGADGKTEVVTIDGKTYNAS : 373
B571_FliC   : GDDIYA-ATKNKDGSISINATEYTDKDGNTKTALNQLGGVDGKTEVVTIDGKTYNAS : 364
B1951_FliC  : GADIYA-ATKNKDGSFSINTTEYTDKDGNTKTALNQLGGADGKTEVVSIDGKTYNAS : 367
B560_FliC   : GDDYYA-ATKEKDGSYSINSTSYTDKDGNTKTALNQLGGADGKTEVVSIDGKTYNAS : 372
B558_FliC   : GDDYYA-ATKEKDGSYSINSTSYTDKDGNTKTALNQLGGADGKTEVVSIDGKTYNAS : 371
                                         G   T

                         *        420         *        440         *
B554_FliC   : KTA-SGVSTLINEDAAAAKKSTANPLASIDSALSKVDAVRSSLGAIQNRFDSAITNL : 433
B2359_FliC  : KTA-SGVSTLINEDAAAAKKSTANPLASIDSALSKVDAVRSSLGAIQNRFDSAITNL : 433
B2486_FliC  : KTA-SGVSTLINEDAAAAKKSTANPLASIDSALSKVDAVRSSLGAIQNRFDSAITNL : 433
B2334_FliC  : KTA-SGVSTLINEDAAAAKKSTANPLASIDSALSKVDAVRSSLGAIQNRFDSAITNL : 433
R160_FliC   : SDVKGHNFQSGGALSEAVTTKTENPLAKIDAALAQVDALRSDLGAVQNRFNSAITNL : 423
B1533_FliC  : SDLDKHNFRTGGELKEVNTDKTENPLQKIDAALAQVDTLRSDLGAVQNRFNSAITNL : 434
B1830_FliC  : SDLDKHNFRTGGELKEVNTDKTENPLQKIDAALAQVDTLRSDLGAVQNRFNSAITNL : 434
B1040_FliC  : KAE-GHNFKAQPDLAEAAATTTENPLQKIDAALAQVDTLRSDLGAVQNRFNSAITNL : 421
B1085_FliC  : KAE-GHNFKAQPDLAEAAATTTENPLQKIDAALAQVDTLRSDLGAVQNRFNSAITNL : 421
B2075_FliC  : KAE-GHNFKAQPDLAEAAATTTENPLQKIDAALAQVDTLRSDLGAVQNRFNSAITNL : 421
B2078_FliC  : KAE-GHNFKAQPDLAEAAATTTENPLQKIDAALAQVDTLRSDLGAVQNRFNSAITNL : 421
B2183_FliC  : KAE-GHNFKAQPDLAEAAATTTENPLQKIDAALAQVDTLRSDLGAVQNRFNSAITNL : 421
B2257_FliC  : KAE-GHNFKAQPDLAEAAATTTENPLQKIDAALAQVDTLRSDLGAVQNRFNSAITNL : 421
B2396_FliC  : KAE-GHNFKAQPDLAEAAATTTENPLQKIDAALAQVDTLRSDLGAVQNRFNSAITNL : 421
xy82_FliC   : KAE-GHNFKAQPDLAEAAATTTENPLQKIDAALAQVDTLRSDLGAVQNRFNSAITNL : 421
R269_FliC   : KAE-GHNFKAQPDLAEAAATTTENPLQKIDAALAQVDTLRSDLGAVQNRFNSAITNL : 423
xy810_FliC  : KAA-GHNFKAQPELAEAAATTTENPLQKIDAALAQVDALRSDLGAVQNRFNSAITNL : 429
B571_FliC   : KAA-GHDFKAQPELAEAAATTTENPLQKIDAALAQVDALRSDLGAVQNRFNSAITNL : 420
B1951_FliC  : KAA-GHNFKAQPELAEAAAATTENPLAKIDAALAQVDALRSDLGAVQNRFNSAITNL : 423
B560_FliC   : KAA-GHNFKAQPDLAEAAATTTENPLQKIDAALAQVDALRSDLGAVQNRFNSAITNL : 428
B558_FliC   : KAA-GHNFKAQPDLAEAAATTTENPLQKIDAALAQVDALRSDLGAVQNRFNSAITNL : 427
                                   T NPL  ID AL  VD 6RS LGA6QNRF1SAITNL

               460         *        480         *        500         *
B554_FliC   : GNTVTNLNSARSRIEDADYATEVSNMSKAQILQQAGTSVLAQANQVPQNVLSLLR* : 488
B2359_FliC  : GNTVTNLNSARSRIEDADYATEVSNMSKAQILQQAGTSVLAQANQVPQNVLSLLR* : 488
B2486_FliC  : GNTVTNLNSARSRIEDADYATEVSNMSKAQILQQAGTSVLAQANQVPQNVLSLLR* : 488
B2334_FliC  : GNTVTNLNSARSRIEDADYATEVSNMSKAQILQQAGTSVLAQANQVPQNVLSLLR* : 488
R160_FliC   : GNTVNNLSEARSRIEDSDYATEVSNMSRAQILQQAGTSVLAQANQVPQNVLSLLR* : 478
B1533_FliC  : GNTVNNLSSARSRIEDSDYATEVSNMSRAQILQQAGTSVLAQANQVPQNVLSLLR* : 489
B1830_FliC  : GNTVNNLSSARSRIEDSDYATEVSNMSRAQILQQAGTSVLAQANQVPQNVLSLLR* : 489
B1040_FliC  : GNTVNNLTSARSRIEDSDYATEVSNMSRAQILQQAGTSVLAQANQVPQNVLSLLR* : 476
B1085_FliC  : GNTVNNLTSARSRIEDSDYATEVSNMSRAQILQQAGTSVLAQANQVPQNVLSLLR* : 476
B2075_FliC  : GNTVNNLTSARSRIEDSDYATEVSNMSRAQILQQAGTSVLAQANQVPQNVLSLLR* : 476
B2078_FliC  : GNTVNNLTSARSRIEDSDYATEVSNMSRAQILQQAGTSVLAQANQVPQNVLSLLR* : 476
B2183_FliC  : GNTVNNLTSARSRIEDSDYATEVSNMSRAQILQQAGTSVLAQANQVPQNVLSLLR* : 476
B2257_FliC  : GNTVNNLTSARSRIEDSDYATEVSNMSRAQILQQAGTSVLAQANQVPQNVLSLLR* : 476
B2396_FliC  : GNTVNNLTSARSRIEDSDYATEVSNMSRAQILQQAGTSVLAQANQVPQNVLSLLR* : 476
xy82_FliC   : GNTVNNLTSARSRIEDSDYATEVSNMSRAQILQQAGTSVLAQANQVPQNVLSLLR* : 476
R269_FliC   : GNTVNNLTSARSRIEDSDYATEVSNMSRAQILQQAGTSVLAQANQVPQNVLSLLR* : 478
xy810_FliC  : GNTVNNLSSARSRIEDSDYATEVSNMSRAQILQQAGTSVLAQANQVPQNVLSLLR* : 484
B571_FliC   : GNTVNNLSSARSRIEDSDYATEVSNMSRAQILQQAGTSVLAQANQVPQNVLSLLR* : 475
B1951_FliC  : GNTVNNLSSARSRIEDSDYATEVSNMSRAQILQQAGTSVLAQANQVPQNVLSLLR* : 478
B560_FliC   : GNTVNNLSSARSRIEDSDYATEVSNMSRAQILQQAGTSVLAQANQVPQNVLSLLR* : 483
B558_FliC   : GNTVNNLSSARSRIEDSDYATEVSNMSRAQILQQAGTSVLAQANQVPQNVLSLLR* : 482
              GNTV NL  ARSRIED DYATEVSNMS4AQILQQAGTSVLAQANQVPQNVLSLLR
```

Continued on next page

Faber *et al.*, Figure S9

Fig. S9: amino acid alignment of FliC flagellin sequences of *Salmonella* isolates used in this study. *fliC* genes of *Salmonella* isolates were amplified using the primers FSa1 and rFSa1 (Dauga *et al.*, 1998) and Sanger-sequenced using the same primers and additionally primer rFSa1_2 (see Suppl. Tab. 7). Derived amino acid sequences (first 17 amino acids not sequenced) were aligned using ClustalOmega (Sievers *et al.*, 2011) and depicted according to their physico-chemical properties using GeneDoc (Nicholas *et al.*, 1997). Strain numbers of strains listed in Table 1 which are included in this alignment but not in Fig. S10 only possess the *fliC* gene (e.g. B571) and served as experimental controls for the strains' FliC flagellin-specific activation potential.

```
                       *        20         *        40         *        60         *
B558_FljB  : NLNKSQSALGTAIERLSSGLRINSAKDDAAGQAIANRFTANIKGLTQASRNANDGISIAQTTEGALNEINNNLQRV : 76
B560_FljB  : NLNKSQSALGTAIERLSSGLRINSAKDDAAGQAIANRFTANIKGLTQASRNANDGISIAQTTEGALNEINNNLQRV : 76
xy82_FljB  : -LNKSQSALGTAIERLSSGLRINSAKDDAAGQAIANRFTANIKGLTQASRNANDGISIAQTTEGALNEINNNLQRV : 75
R269_FljB  : -LNKSQSALGTAIERLSSGLRINSAKDDAAGQAIANRFTANIKGLTQASRNANDGISIAQTTEGALNEINNNLQRV : 75
R160_FljB  : NLNKSQSALGTAIERLSSGLRINSAKDDAAGQAIANRFTANIKGLTQASRNANDGISIAQTTEGALNEINNNLQRV : 76
xy810_FljB : NLNKSQSALGTAIERLSSGLRINSAKDDAAGQAIANRFTANIKGLTQASRNANDGISIAQTTEGALNEINNNLQRV : 76
B815_FljB  : -LNKSQSALGTAIERLSSGLRINSAKDDAAGQAIANRFTANIKGLTQASRNANDGISIAQTTEGALNEINNNLQRV : 75
B1951_FljB : -LNKSQSALGTAIERLSSGLRINSAKDDAAGQAIANRFTANIKGLTQASRNANDGISIAQTTEGALNEINNNLQRV : 75
B1040_FljB : -LNKSQSALGTAIERLSSGLRINSAKDDAAGQAIANRFTANIKGLTQASRNANDGISIAQTTEGALNEINNNLQRV : 75
B1085_FljB : -LNKSQSALGTAIERLSSGLRINSAKDDAAGQAIANRFTANIKGLTQASRNANDGISIAQTTEGALNEINNNLQRV : 75
B2078_FljB : -LNKSQSALGTAIERLSSGLRINSAKDDAAGQAIANRFTANIKGLTQASRNANDGISIAQTTEGALNEINNNLQRV : 75
B2183_FljB : -LNKSQSALGTAIERLSSGLRINSAKDDAAGQAIANRFTANIKGLTQASRNANDGISIAQTTEGALNEINNNLQRV : 75
B2257_FljB : -LNKSQSALGTAIERLSSGLRINSAKDDAAGQAIANRFTANIKGLTQASRNANDGISIAQTTEGALNEINNNLQRV : 75
B2396_FljB : -LNKSQSALGTAIERLSSGLRINSAKDDAAGQAIANRFTANIKGLTQASRNANDGISIAQTTEGALNEINNNLQRV : 75
B1050_FljB : -LNKSQSALGTAIERLSSGLRINSAKDDAAGQAIANRFTANIKGLTQASRNANDGISIAQTTEGALNEINNNLQRV : 75
xy92_FljB  : -LNKSQSALGTAIERLSSGLRINSAKDDAAGQAIANRFTANIKGLTQASRNANDGISIAQTTEGALNEINNNLQRV : 75
2075_FljB  : -LNKSQSALGTAIERLSSGLRINSAKDDAAGQAIANRFTANIKGLTQASRNANDGISIAQTTEGALNEINNNLQRV : 75
              LNKSQSALGTAIERLSSGLRINSAKDDAAGQAIANRFTANIKGLTQASRNANDGISIAQTTEGALNEINNNLQRV

               80         *       100         *       120         *       140         *
B558_FljB  : RELAVQSANSTNSQSDLDSIQAEITQRLNEIDRVSGQTQFNGVKVLAQDNTLTIQVGANDGETIDIDLKQINSQTL : 152
B560_FljB  : RELAVQSANSTNSQSDLDSIQAEITQRLNEIDRVSGQTQFNGVKVLAQDNTLTIQVGANDGETIDIDLKQINSQTL : 152
xy82_FljB  : RELAVQSANSTNSQSDLDSIQAEITQRLNEIDRVSGQTQFNGVKVLAQDNTLTIQVGANDGETIDIDLKQINSQTL : 151
R269_FljB  : RELAVQSANSTNSQSDLDSIQAEITQRLNEIDRVSGQTQFNGVKVLAQDNTLTIQVGANDGETIDIDLKQINSQTL : 151
R160_FljB  : RELAVQSANSTNSQSDLDSIQAEITQRLNEIDRVSGQTQFNGVKVLAQDNTLTIQVGANDGETIDIDLKQINSQTL : 152
xy810_FljB : RELAVQSANSTNSQSDLDSIQAEITQRLNEIDRVSGQTQFNGVKVLAQDNTLTIQVGANDGETIDIDLKQINSQTL : 152
B815_FljB  : RELAVQSANSTNSQSDLDSIQAEITQRLNEIDRVSGQTQFNGVKVLAQDNTLTIQVGANDGETIDIDLKQINSQTL : 151
B1951_FljB : RELAVQSANSTNSQSDLDSIQAEITQRLNEIDRVSGQTQFNGVKVLAQDNTLTIQVGANDGETIDIDLKQINSQTL : 151
B1040_FljB : RELAVQSANSTNSQSDLDSIQAEITQRLNEIDRVSGQTQFNGVKVLAQDNTLTIQVGANDGETIDIDLKQINSQTL : 151
B1085_FljB : RELAVQSANSTNSQSDLDSIQAEITQRLNEIDRVSGQTQFNGVKVLAQDNTLTIQVGANDGETIDIDLKQINSQTL : 151
B2078_FljB : RELAVQSANSTNSQSDLDSIQAEITQRLNEIDRVSGQTQFNGVKVLAQDNTLTIQVGANDGETIDIDLKQINSQTL : 151
B2183_FljB : RELAVQSANSTNSQSDLDSIQAEITQRLNEIDRVSGQTQFNGVKVLAQDNTLTIQVGANDGETIDIDLKQINSQTL : 151
B2257_FljB : RELAVQSANSTNSQSDLDSIQAEITQRLNEIDRVSGQTQFNGVKVLAQDNTLTIQVGANDGETIDIDLKQINSQTL : 151
B2396_FljB : RELAVQSANSTNSQSDLDSIQAEITQRLNEIDRVSGQTQFNGVKVLAQDNTLTIQVGANDGETIDIDLKQINSQTL : 151
B1050_FljB : RELAVQSANSTNSQSDLDSIQAEITQRLNEIDRVSGQTQFNGVKVLAQDNTLTIQVGANDGETIDIDLKQINSQTL : 151
xy92_FljB  : RELAVQSANSTNSQSDLDSIQAEITQRLNEIDRVSGQTQFNGVKVLAQDNTLTIQVGANDGETIDIDLKQINSQTL : 151
2075_FljB  : RELAVQSANSTNSQSDLDSIQAEITQRLNEIDRVSGQTQFNGVKVLAQDNTLTIQVGANDGETIDIDLKQINSQTL : 151
             RELAVQSANSTNSQSDLDSIQAEITQRLNEIDRVSGQTQFNGVKVLAQDNTLTIQVGANDGETIDIDLKQINSQTL

                  160         *       180         *       200         *       220
B558_FljB  : GLDSLNVQKAYDVSATDVISSTYSDGTQALTAPTA--TDIKAALGNPTVTGDTLTAAV--SFKDGKYYATVSGYTD : 224
B560_FljB  : GLDSLNVQKAYDVSATDVISSTYSDGTQALTAPTA--TDIKAALGNPTVTGDTLTAAV--SFKDGKYYATVSGYTD : 224
xy82_FljB  : GLDSLNVQKAYDVKDTAVTTKAYADNGTTLDASGLDDAAIKAAIGGTTGTAAVTGGTVKFDADNNKYFVTIGGFTG : 227
R269_FljB  : GLDSLNVQKAYDVKDTAVTTKAYANNGTTLDVSGLDDAAIKAATGGTNGTASVTGGAVKFDADNNKYFVTIGGFTG : 227
R160_FljB  : GLDSLNVQKAYDVKDTAVTTKAYANNGTTLDVSGLDDAAIKAATGGTNGTASVTGGAVKFDADNNKYFVTIGGFTG : 228
xy810_FljB : GLDSLNVQKAYDVKDTAVTTKAYANNGTTLDVSGLDDTAIKAAIGGTTGTAAVTGSAVKFDADNNKYFVTIGGFTG : 228
B815_FljB  : GLDSLNVQKAYDVKDTAVTTKAYANNGTTLDVSGLDDTAIKAAIGGTTGTAAVTGSAVKFDADNNKYFVTIGGFTG : 227
B1951_FljB : GLDSLNVQKAYDVKDTAVTTKAYADNGTTLDASGLDDAAIKAAIGGTTGTAAVTSGTVKFDADNNKYFVTIGGFTG : 227
B1040_FljB : GLDSLNVQKAYDVKDTAVTTTAYADNGTTLDVSGLTDAAIKTATGGTTGTASVTGGAVKFDADNNKYFVTIGGFTG : 227
B1085_FljB : GLDSLNVQKAYDVKDTAVTTTAYADNGTTLDVSGLTDAAIKTATGGTTGTASVTGGAVKFDADNNKYFVTIGGFTG : 227
B2078_FljB : GLDSLNVQKAYDVKDTAVTTTAYADNGTTLDVSGLTDAAIKTATGGTTGTASVTGGAVKFDADNNKYFVTIGGFTG : 227
B2183_FljB : GLDSLNVQKAYDVKDTAVTTTAYADNGTTLDVSGLTDAAIKTATGGTTGTASVTGGAVKFDADNNKYFVTIGGFTG : 227
B2257_FljB : GLDSLNVQKAYDVKDTAVTTTAYADNGTTLDVSGLTDAAIKTATGGTTGTASVTGGAVKFDADNNKYFVTIGGFTG : 227
B2396_FljB : GLDSLNVQKAYDVKDTAVTTTAYADNGTTLDVSGLTDAAIKTATGGTTGTASVTGGAVKFDADNNKYFVTIGGFTG : 227
B1050_FljB : GLDSLNVQKAYDVKDTAVTTTAYADNGTTLDVSGLTDAAIKTATGGTTGTASVTGGAVKFDADNNKYFVTIGGFTG : 227
xy92_FljB  : GLDSLNVQKAYDVKDTAVTTTAYADNGTTLDVSGLTDAAIKTATGGTTGTASVTGGAVKFDADNNKYFVTIGGFTG : 227
2075_FljB  : GLDSLNVQKAYDVKDTAVTTTAYADNGTTLDVSGLTDAAIKTATGGTTGTASVTGGAVKFDADNNKYFVTIGGFTG : 227
             GLDSLNVQKAYDVkdTaVt3 aYa1ngttLd sgl d aIK A GgttgTa vtg aVkfdad1nKY5vT6gG5Tg
```

Continued on next page

```
                 *       240         *       260         *       280         *       300
B558_FljB   : AGDTAKNGKYEVTVDSATGAVSFGATPTKSTVT-GDTAVTK--VQVNAPVAADAATKKALQDGGVSSADASAATLV : 297
B560_FljB   : AGDTAKNGKYEVTVDSATGAVSFGATPTKSTVT-GDTAVTK--VQVNAPVAADAATKKALQDGGVSSADASAATLV : 297
xy82_FljB   : A-DAAKNGDYEVNVATD-GKVTLATGATKTTMPAGAATKTEXQELKDTPAVVSADAKNALIAGGVDTADANAATLV : 301
R269_FljB   : A-DAAKNGDYEVNVATD-GTVTLAAGATKTTMPAGATTKTEVQELKDTPAVVSADAKNALIAGGVDATDANGAELV : 301
R160_FljB   : A-DAAKNGDYEVNVATD-GTVTLAAGATKTTMPAGATTKTEVQELKDTPAVVSADAKNALIAGGVDATDANGAELV : 302
xy810_FljB  : A-DAAKNGDYEVNVATD-GTVTLAAGATKTTMPAGATTKTEVQELKDTPAVVSADAKNALIAGGVDATDANGAELV : 302
B815_FljB   : A-DAAKNGDYEVNVATD-GTVTLAAGATKTTMPAGATTKTEVQELKDTPAVVSADAKNALIAGGVDATDANGAELV : 301
B1951_FljB  : A-DAAKNGDYEVNVATD-GKVTLATSATKTTMPAGAATKTEVQELKDTPAVVSADAKNALIAGGVDTADANAATLV : 301
B1040_FljB  : A-DAAKNGDYEVNVATD-GTVTLAAGATKTTMPAGVTTKTEVQELTTTPVVASADAKNALIAGGVDTADANAATLV : 301
B1085_FljB  : A-DAAKNGDYEVNVATD-GTVTLAAGATKTTMPAGVTTKTEVQELTTTPVVASADAKNALIAGGVDTADANAATLV : 301
B2078_FljB  : A-DAAKNGDYEVNVATD-GTVTLAAGATKTTMPAGVTTKTEVQELTTTPVVASADAKNALIAGGVDTADANAATLV : 301
B2183_FljB  : A-DAAKNGDYEVNVATD-GTVTLAAGATKTTMPAGVTTKTEVQELTTTPVVASADAKNALIAGGVDTADANAATLV : 301
B2257_FljB  : A-DAAKNGDYEVNVATD-GTVTLAAGATKTTMPAGVTTKTEVQELTTTPVVASADAKNALIAGGVDTADANAATLV : 301
B2396_FljB  : A-DAAKNGDYEVNVATD-GTVTLAAGATKTTMPAGVTTKTEVQELTTTPVVASADAKNALIAGGVDTADANAATLV : 301
B1050_FljB  : A-DAAKNGDYEVNVATD-GTVTLAAGATKTTMPAGVTTKTEVQELTTTPVVASADAKNALIAGGVDTADANAATLV : 301
xy92_FljB   : A-DAAKNGDYEVNVATD-GTVTLAAGATKTTMPAGVTTKTEVQELTTTPVVASADAKNALIAGGVDTADANAATLV : 301
2075_FljB   : A-DAAKNGDYEVNVATD-GTVTLAAGATKTTMPAGVTTKTEVQELTTTPVVASADAKNALIAGGVDTADANAATLV : 301
              A DaAKNGdYEVnVa3d G V3laagaTK3T6paG ttkTevqel  tP v sAdaKnALiaGGVd  DAn A LV

                    *       320         *       340         *       360         *       380
B558_FljB   : KMSYTDKNGKTIEGGYALKAGDKYYAADYDEATGAIKAKTTSYTAADGTTKTAANQLGGVDGKTEVVTIDGKTYNA : 373
B560_FljB   : KMSYTDKNGKTIEGGYALKAGDKYYAADYDEATGAIKAKTTSYTAADGTTKTAANQLGGVDGKTEVVTIDGKTYNA : 373
xy82_FljB   : KMXYTDKNGXTIEGGYALKAGDKYYAADYDEATGAIKAKTTSYTAADGTTKTAANQLGGVDGKTEVVTIDGKTYNA : 377
R269_FljB   : KMSYTDKNGKTIEGGYALKAGDKYYAADYDEATGAIKAKTTSYTAADGTTKTAANQLGGVDGKTEVVTIDGKTYNA : 377
R160_FljB   : KMSYTDKNGKTIEGGYALKAGDKYYAADYDEATGAIKAKTTSYTAADGTTKTAANQLGGVDGKTEVVTIDGKTYNA : 378
xy810_FljB  : KMSYTDKNGKTIEGGYALKAGDKYYAADYDEATGAIKAKTTSYTAADGTTKTAANQLGGVDGKTEVVTIDGKTYNA : 378
B815_FljB   : KMSYTDKNGKTIEGGYALKAGDKYYAADYDEATGAIKAKTTSYTAADGTTKTAANQLGGVDGKTEVVTIDGKTYNA : 377
B1951_FljB  : KMSYTDKNGKTIEGGYALKAGDKYYAADYDEATGAIKAKTTSYTAADGTTKTAANQLGGVDGKTEVVTIDGKTYNA : 377
B1040_FljB  : KMSYTDKNGKTIEGGYALKAGDKYYAADYDEATGAIKAKTTSYTAADGTTKTAANQLGGVDGKTEVVTIDGKTYNA : 377
B1085_FljB  : KMSYTDKNGKTIEGGYALKAGDKYYAADYDEATGAIKAKTTSYTAADGTTKTAANQLGGVDGKTEVVTIDGKTYNA : 377
B2078_FljB  : KMSYTDKNGKTIEGGYALKAGDKYYAADYDEATGAIKAKTTSYTAADGTTKTAANQLGGVDGKTEVVTIDGKTYNA : 377
B2183_FljB  : KMSYTDKNGKTIEGGYALKAGDKYYAADYDEATGAIKAKTTSYTAADGTTKTAANQLGGVDGKTEVVTIDGKTYNA : 377
B2257_FljB  : KMSYTDKNGKTIEGGYALKAGDKYYAADYDEATGAIKAKTTSYTAADGTTKTAANQLGGVDGKTEVVTIDGKTYNA : 377
B2396_FljB  : KMSYTDKNGKTIEGGYALKAGDKYYAADYDEATGAIKAKTTSYTAADGTTKTAANQLGGVDGKTEVVTIDGKTYNA : 377
B1050_FljB  : KMSYTDKNGKTIEGGYALKAGDKYYAADYDEATGAIKAKTTSYTAADGTTKTAANQLGGVDGKTEVVTIDGKTYNA : 377
xy92_FljB   : KMSYTDKNGKTIEGGYALKAGDKYYAADYDEATGAIKAKTTSYTAADGTTKTAANQLGGVDGKTEVVTIDGKTYNA : 377
2075_FljB   : KMSYTDKNGKTIEGGYALKAGDKYYAADYDEATGAIKAKTTSYTAADGTTKTAANQLGGVDGKTEVVTIDGKTYNA : 377
              KMsYTDKNGkTIEGGYALKAGDKYYAADYDEATGAIKAKTTSYTAADGTTKTAANQLGGVDGKTEVVTIDGKTYNA

                         *       400         *       420         *       440         *
B558_FljB   : SKAAGHDFKAQPELAEAAAKTTENPLQKIDAALAQVDALRSDLGAVQNRFNSAITNLGNTVNNLSEARSRIEDSDY : 449
B560_FljB   : SKAAGHDFKAQPELAEAAAKTTENPLQKIDAALAQVDALRSDLGAVQNRFNSAITNLGNTVNNLSEARSRIEDSDY : 449
xy82_FljB   : SKAAGHDFKAQPELAEAAAKTTENPLQKIDAALAQVDALRSDLGAVQNRFNSAITNLGNTVNNLSEARSRIEDSDY : 453
R269_FljB   : SKAAGHDFKAQPELAEAAAKTTENPLQKIDAALAQVDALRSDLGAVQNRFNSAITNLGNTVNNLSEARSRIEDSDY : 453
R160_FljB   : SKAAGHDFKAQPELAEAAAKTTENPLQKIDAALAQVDALRSDLGAVQNRFNSAITNLGNTVNNLSEARSRIEDSDY : 454
xy810_FljB  : SKAAGHDFKAQPELAEAAAKTTENPLQKIDAALAQVDALRSDLGAVQNRFNSAITNLGNTVNNLSEARSRIEDSDY : 454
B815_FljB   : SKAAGHDFKAQPELAEAAAKTTENPLQKIDAALAQVDALRSDLGAVQNRFNSAITNLGNTVNNLSEARSRIEDSDY : 453
B1951_FljB  : SKAAGHDFKAQPELAEAAAKTTENPLQKIDAALAQVDALRSDLGAVQNRFNSAITNLGNTVNNLSEARSRIEDSDY : 453
B1040_FljB  : SKAAGHDFKAQPELAEAAAKTTENPLQKIDAALAQVDALRSDLGAVQNRFNSAITNLGNTVNNLSEARSRIEDSDY : 453
B1085_FljB  : SKAAGHDFKAQPELAEAAAKTTENPLQKIDAALAQVDALRSDLGAVQNRFNSAITNLGNTVNNLSEARSRIEDSDY : 453
B2078_FljB  : SKAAGHDFKAQPELAEAAAKTTENPLQKIDAALAQVDALRSDLGAVQNRFNSAITNLGNTVNNLSEARSRIEDSDY : 453
B2183_FljB  : SKAAGHDFKAQPELAEAAAKTTENPLQKIDAALAQVDALRSDLGAVQNRFNSAITNLGNTVNNLSEARSRIEDSDY : 453
B2257_FljB  : SKAAGHDFKAQPELAEAAAKTTENPLQKIDAALAQVDALRSDLGAVQNRFNSAITNLGNTVNNLSEARSRIEDSDY : 453
B2396_FljB  : SKAAGHDFKAQPELAEAAAKTTENPLQKIDAALAQVDALRSDLGAVQNRFNSAITNLGNTVNNLSEARSRIEDSDY : 453
B1050_FljB  : SKAAGHDFKAQPELAEAAAKTTENPLQKIDAALAQVDALRSDLGAVQNRFNSAITNLGNTVNNLSEARSRIEDSDY : 453
xy92_FljB   : SKAAGHDFKAQPELAEAAAKTTENPLQKIDAALAQVDALRSDLGAVQNRFNSAITNLGNTVNNLSEARSRIEDSDY : 453
2075_FljB   : SKAAGHDFKAQPELAEAAAKTTENPLQKIDAALAQVDALRSDLGAVQNRFNSAITNLGNTVNNLSEARSRIEDSDY : 453
              SKAAGHDFKAQPELAEAAAKTTENPLQKIDAALAQVDALRSDLGAVQNRFNSAITNLGNTVNNLSEARSRIEDSDY
```

Continued on next page

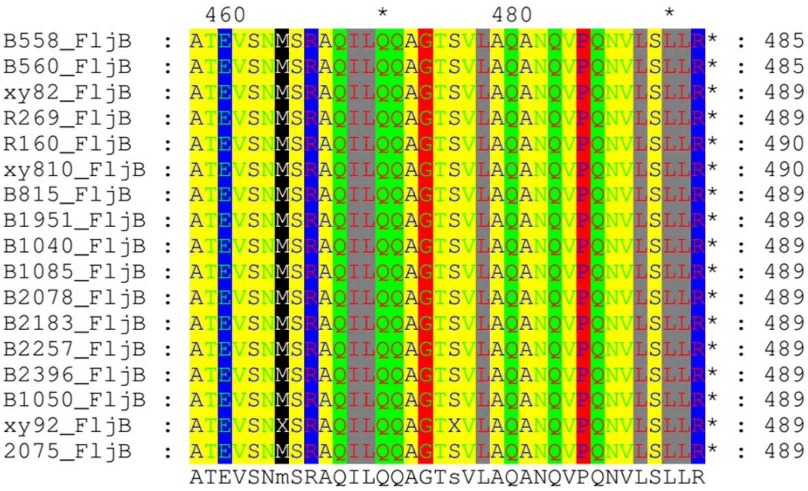

Faber *et al.*, Figure S10

Fig. S10: amino acid alignment of FljB flagellin sequences of *Salmonella* isolates used in this study. *fljB* genes of *Salmonella* isolates were amplified using the primers FSa2 and rFSa2 (Dauga *et al*., 1998) and Sanger-sequenced using the same primers and additionally primer rFSa1_3 (see Suppl. Tab 7). Derived amino acid sequences (first 16 or 17 amino acids not sequenced) were aligned with ClustalOmega (Sievers *et al*., 2011) and depicted according to their physico-chemical properties using GeneDoc (Nicholas *et al*., 1997). Strain numbers of strains listed in Table 1 which are included in this alignment but not in Fig. S9 only possess the *flgJ* gene (e.g. B815) and served as controls for the strains' FlgJ flagellin-specific activation potential.

```
chTLR5      ------------mmlhqrliivfg-ialagdicasrscysevqvsmynscnltgvppvpk    47
mTLR5       mdaefphaphfsrimacqldlligvifmaspvlvispcssdgriaffrgcnltqipwiln    60
hTLR5       --------------mgdhldlllgvvlmagpvfgipscsfdgriafyrfcnltqvpqvln    46
bTLR5       --------------mgdcldlllgvvlltspalgmsscffdgwraiylscnltqvpqvpn    46
pTLR5       --------------mgdclvlllt-llvaspalgmpscffdgqraiyrgcnltqvpqvps    45
                          :   * :::  : ::.        *  :    :::  **** :* : .

chTLR5      d-taklfltynyirqvtatsfplledlflleigtqrvfplyigkeafrnlpnlrvldlgf    106
mTLR5       ttterlllsfnyismvvatsfpllerlqllelgtqy-anltigpgafrnlpnlrildlgq    119
hTLR5       t-terlllsfnyirtvtassfpfleqlqllelgsqy-tpltidkeafrnlpnlrildlgs    104
bTLR5       t-tkslllsfnyirtvttasfpfleqlqllelgtqf-tpltiyreafrnlpnlrildlgg    104
pTLR5       g-tkslllsfnyirtvtagsfpfleglqllelgtql-tplsidreafrnlpnlrildlgh    103
              *  *:*::***  *.: ***:** * ***:*:*    * *   *********:****

chTLR5      nnilllddldsfaglqrltilrlfqnnlgdsileeryfqdlrsleeldlsgnqitklhphp    166
mTLR5       sqievlnrdafqglphllelrlfscglssavlsdgyfrnlyslarldlsgnqihslrlhs    179
hTLR5       skiyflhpdafqglfhlfelrlyfcglsdavlkdgyfrnlkaltrldlsknqirslylhp    164
bTLR5       sqinflhpdafqglphltklrlfscglsdavlkdgyfrnlaslthldlsknkiqslylhp    164
pTLR5       sqiaflhpdafqelphlselrlfscglsdailkdgyfrnlasltrldlsknqiqslhlhp    163
            .:* .*. *:*  * :*  ***:  .*..::*.: **::* :* .**** *:* .*  *

chTLR5      lfynltilkavnlkfnkisnlcegnltsfqgkhfsffslstntlyktdkmiwakcpnpfr    226
mTLR5       sfrelnslsdvnfafnqifticedeleplqgktlsffglkltklfsrvsvgwetcrnpfr    239
hTLR5       sfgklnslksidfssnqiflvceheleplqgktlsffslaanslysrvsvdwgkcmnpfr    224
bTLR5       sfrelnslksidfsfnkipivceqefkplqgktlsflsladnqlysrvsvdwnkclnpfr    224
pTLR5       sfqelnslkaidlslnqiplvceqglkplqgktlsflsladnnlysrvsvdwgkcmnpfr    223
             * :*. *. :::  *:*  :**  :  :*** :**:.*  . *:.  .: * .* ****

chTLR5      nitfnsldvsengwstetvqyfctaikgtqinylsfrshtmgsgfgfnnlknpdtdtftg    286
mTLR5       gvrletldlsengwtvditrnfsniiqgsqisslilkhhimgpgfgfqnirdpdqstfas    299
hTLR5       nmvleildvsgngwtvditgnfsnaisksqafslilahhimgagfgfhnikdpdqntfag    284
bTLR5       nmvletldvsgngwgvdimrnfsnaingsqifslvltrhimgssfgfsnlkdpdyhtfag    284
pTLR5       nmalemldvsgngwtadttrnfsqavngsqisslvlahhimgsgfgfhnikdpdhhtfas    283
            .: :: **:* *** .:    *.  :. :*   * :  * ** .*** *:::**  **:.

chTLR5      larsdlhlldisngfifslnslifeslrnleflnlfrnkinqiqkqaffglenleilnls    346
mTLR5       larssvlqldlshgfifslnprlfgtlkdlkmlnlafnkinkigenafygldslqvlnls    359
hTLR5       larssvrhldlshgfvfslnsrvfetlkdlkvlnlaynkinkiadeafygldnlqvlnls    344
bTLR5       larssmiqldishgyifslnfrifetlqelkvlnlaynkinsisrnafygldnlqvlnis    344
pTLR5       lgrssliqldlshgfifslnfrlfgtlkelkvlnlafnkinkiadqafhgldnlqilnms    343
            *.**.:  **:*:*::****  :* :*::*:.***  ****.*  :**.**:.*::**:*

chTLR5      snllgelydytfeglhsimyidlqqnhiqmigeksfsnlvnlkiidlrdnaikklpsfph    406
mTLR5       ynllgelynsnfyglprvayvdlqrnhigiiqdqtfrllktlqtldlrdnalkaigfips    419
hTLR5       ynllgelyssnfyglpkvayidlqknhiaiiqdqtfkfleklqtldlrdnalttihfips    404
bTLR5       ynllgelynydfdglpkvayidlqknhigiiqdktfkflgklntldlrdnalktiyflps    404
pTLR5       ynllgelynsnfeglpklayidlqknhigiiqdqtfrflkklntldlrdnalktiqfips    403
             *******.  * **  : *:***:***.:* :::*  * .*: :******:. :  :*

chTLR5      ltsaflsdnklmsvahtaivathielernwlanlgdlyvlfqvpgvqylllkqnrfsycv    466
mTLR5       iqmvllggnklvhlphihftanflelsenrlenlsdlyfllrvpqlqflilnqnrlssck    479
hTLR5       ipdiflsgnklvtlpkinltanlihlsenrlenldilyfllrvphlqililnqnrfsscs    464
bTLR5       ipniflsgnklmtlpnipltanfiqlsenrlenlndlyfllqvphlqililnqnrfsfch    464
pTLR5       iptlflggnklvtlpnirltanfihlsenrlenldnlyfllqvphlqililnqnrfsncn    463
            :   :*..***: : :  :.*. :.*..* * **. **.*::** :* *:*:***:* *

chTLR5      khvdaiennqliymdlgenmlqlvwerglcldvfrtlsklqvlhlnnnylsalpqeifng    526
mTLR5       aahtpsenpsleqlfltenmlqlawetglcwdvfqglsrlqilylsnnylnflppgifnd    539
hTLR5       gdqtpsenpsleqlflgenmlqlawetelcwdvfeglshlqvlylnhnylnslppgvfsh    524
bTLR5       qnhapsensslekflfgenmlqlawetgscwdifkglshlqllylnknylnflppgvfhh    524
pTLR5       qrhapsenpsleqlflgenmlqlaweagfcwdvfkglshlqvlylnnnylnflppgvfrh    523
                  ** .*  : * ******.**   * *:*. **:**:*:*.:***. **  :*

chTLR5      ltslkrlnlasnllshlslrvfpqslinlnlsgnqlfspkpkvfmtlsilldithnkyvcd    586
mTLR5       lvalrmlslsankltvlspgslpanleildisrnqlfspdpalfsslrvldithnefvcn    599
hTLR5       ltalrglslnsnrltvlshndlpanleildisrnqllapnpdvfvslsvldithnkfice    584
bTLR5       ltalrglslkdnrltvlfpgdlpanleildisgnqllspdpdlfaslsaidithnnfice    584
pTLR5       ltalrglslsynrltvlypgdlpahlevldlsrnqllspdpdlftslsavdlshnkfvcd    583
            *.:*: *.*  * *: *    :*  *  *::* ***::*.* :* :*  :*::**:::*:

chTLR5      calksllvwlnetnvtlagsesdrycvyppalagvpvsfltyddcdedelqqtlrfsvfv    646
mTLR5       celstfiswlnqtnvtlfgspadvycmypnsllggslynistedcdeeeamrslkfslfi    659
hTLR5       celstfinwlnhtnvtiagppadiycvypdsfsgvslfslstegcdeeevlkslkfslfi    644
bTLR5       celsafihwlnqtnitiagspadmycmypnslagvsiyslstesceeeevleslkfslfi    644
pTLR5       celstfinwlnqtnvtifgsrddiycmypsslagsplntvsmagcseeevleslkfslfi    643
            * *.::: ***.**:*: *   * **:** :: *  :  ::  .*.*:*  .:*:**:*:
```

Continued on next page

```
chTLR5      flsvtllmflmstiiftrcrgicfvwyktitktligshppaadtseymydaylcyskndf    706
mTLR5       lctvtltlflvitlvvikfrgicflcyktiqklvfkdkvwslepgayrydayfcfsskdf    719
hTLR5       vctvtltlflmtiltvtkfrgfcficyktaqrlvfkdhpqgtepdmykydaylcfsskdf    704
bTLR5       lvtvtltlflvitlavtkfrgfcficykkaqsllfkdpikgresdtykydaylcfsskdf    704
pTLR5       lftvtltllfvailvvmkfrgfcficykkvqrlvfkdpslgresdtykydaylcfsgkdf    703
            . :*** ::::  : . : **:**: **.    :: .   . : . * ****:*:* :**

chTLR5      ewvqnsllkhldsqyfdknrftlcfeerdflpgeehinnirdaiwksrkticvvtrqflk    766
mTLR5       ewaqnallkhldahyssrnrlrlcfeerdfipgenhisniqaavwgsrktvclvsrhflr    779
hTLR5       twvqnallkhldtqysdqnrfnlcfeerdfvpgenrianiqdaiwnsrkivclvsrhflr    764
bTLR5       ewvqnall-hldvqyhsqnrfnlcfeerdfmpgenhianiqdavwssrkivclvsrhflr    763
pTLR5       ewvqeallknldaqysdqnrlnlcfeerdfvpgedrisniqdavwssrkvvclvsrhflr    763
             *.*::** :** :* .:**: ********:***::* **: *:* *** :*:*:*:**:

chTLR5      dgwcveafnfaqsryfsdlkevlimvvvgslsqyqlmkhkpiriflqrsrylrwpedyqd    826
mTLR5       dgwcleafryaqsrslsdlksilivvvvgslsqyqlmrhetirgflqkqqylrwpedlqd    839
hTLR5       dgwcleafsyaqgrclsdlnsalimvvvgslsqyqlmkhqsirgfvqkqqylrwpedfqd    824
bTLR5       dgwcleafsyaqsrcladlngalimvvvgslsqfhlmrhqsirgfvqkrqylrwpedlqd    823
pTLR5       dgwcleafsyaqsrcladlngtlimvvvgslpqyqlmkhqsirgfvrkqqylrwpedlqd    823
            ****:*** :**.* ::**:  **:****** *::**:*: ** *::: :******* **

chTLR5      igwfldnlssqilkekkvqrnvsgielqtiatvsh 861
mTLR5       vgwfldklsgcilkeekakkrsssiqlrtiatis- 873
hTLR5       vgwflhklsqqilkkekekkkdnniplqtvatis- 858
bTLR5       vdwflnklsqcilkkekerkkdsaiqlqnvttis- 857
pTLR5       vswflnklsqyilkkvkekkkdsdiqlqsv-tis- 856
            :.***.:**  ***: * ::. . * *:.::
```

Faber *et al*., Figure S11

Fig. S11: amino acid alignment of all full-length TLR5 proteins expressed from pEF6-V5 constructs and used for activation in this study. chTLR5 = *Gallus gallus* TLR5; m = *Mus musculus* TLR5; hTLR5 = *Homo sapiens* TLR5; bTLR5 = *Bos taurus* TLR5; pTLR5 = *Sus scrofa* TLR5. Symbols below the alignment designate the level of amino acid (aa) identity or conservation at each site: asterisk = identical aa; two dots = highly conserved aa; one dot = moderately conserved aa. Nucleotide sequences of each cloned insert were determined by Sanger sequencing and translated into amino acid sequences. The alignment was prepared using ClustalOmega (Sievers *et al*., 2011).

Supplementary Tables

Table S1: *Campylobacter jejuni* or *Salmonella enterica* isolates used in this study.

Bacterial isolate	Isolate designation	Isolated from	Host species
Campylobacter jejuni (ST-50)	68	Human feces	Humans, poultry, swine, cattle, sheep, cats, dogs
Campylobacter jejuni (ST-45)	69	Human feces	s. a.
Campylobacter jejuni (ST-50)	70	Human feces	s. a.
Campylobacter jejuni (ST-21)	71	Human feces	s. a.
Campylobacter jejuni (ST-50)	72	Human feces	s. a.
Campylobacter jejuni (ST-21)	73	Human feces	s. a.
Campylobacter jejuni (ST-45)	75	Human feces	s. a.
Campylobacter jejuni (ST-21)	87	Human feces	s. a.
Campylobacter jejuni (ST-21)	89	Human feces	s. a.
Campylobacter jejuni (ST-21)	90	Human feces	s. a.
Campylobacter jejuni ATCC 700819	NCTC 11168	Human feces	s. a.
Campylobacter jejuni (ST-21)	FBI 04321 [A]	Aviary bird	s. a.
Campylobacter jejuni (ST-21)	FBI 04371 [A]	Sheep	s. a.
Campylobacter jejuni (ST-21)	1	Broiler	s. a.
Campylobacter jejuni (ST-21)	FBI 04197 [A]	Cattle feces	s. a.
Campylobacter jejuni (ST-21)	FBI 02626 [A]	Broiler	s. a.
Campylobacter jejuni (ST-21)	FBI 04199 [A]	Cattle feces	s. a.
Salmonella enterica serovar Bovismorbificans	xy 82	n. s.	Cattle, horses
Salmonella enterica serovar Choleraesuis	B 815	Human blood	Swine
Salmonella enterica serovar Choleraesuis	xy 810	n. s.	s. a.
Salmonella enterica serovar Typhimurium	RV 269	n. s.	Humans, cattle, swine, sheep, horses, rodents, poultry
Salmonella enterica serovar Enteritidis	B 554	Human feces	Humans, rodents, poultry, horses
Salmonella enterica serovar Enteritidis	B 558	Human feces	s. a.
Salmonella enterica serovar Enteritidis	B 560	Human feces	s. a.
Salmonella enterica serovar Enteritidis	B 571	Human feces	s. a.
Salmonella enterica serovar Enteritidis	B 2334	Human blood	s. a.
Salmonella enterica serovar Enteritidis	B 2359	Human feces	s. a.
Salmonella enterica serovar Enteritidis	B 2486	Human feces	s. a.
Salmonella enterica serovar Infantis	B 1040	Human urine	Humans, poultry
Salmonella enterica serovar Infantis	B 1050	Human feces	s. a.
Salmonella enterica serovar Infantis	B 1085	Human feces	s. a.
Salmonella enterica serovar Infantis	B 2075	Human feces	s. a.
Salmonella enterica serovar Infantis	B 2078	Human feces	s. a.
Salmonella enterica serovar Infantis	B 2183	n. s.	s. a.
Salmonella enterica serovar Infantis	B 2257	n. s.	s. a.
Salmonella enterica serovar Infantis	B 2396	Human feces	s. a.
Salmonella enterica serovar Infantis	xy 92	n. s.	s. a.
Salmonella enterica serovar Paratyphi A	B 1951	Human blood	Humans
Salmonella enterica serovar Paratyphi B	RV 160	n. s.	Humans

Salmonella enterica serovar Typhi	B	1533	Human feces	Humans
Salmonella enterica serovar Typhi	B	1830	Human blood	s. a.

ST: sequence type
n. s.: not specified
s. a.: see above
[A]: *C. jejuni* isolates published in (Gripp *et al.*, 2011)

Table S2: Plasmids used in this study.

Plasmid	Vector	Description	Resistance	Reference or Source
	pEF6-V5	mammalian protein expression vector, originated from pEF6-TLR5-V5 by excision of the insert and removal of His-tag, EF-1α promoter, V5-tag	Amp^R	(Lee *et al.*, 2003)
pEF6-hTLR5	pEF6-V5	protein expression plasmid for human TLR5; removal of His-tag	Amp^R	(Smith, Jr. *et al.*, 2003; Lee *et al.*, 2003)
pCJ800	pEF6-V5	protein expression plasmid for mouse TLR5 (pEF6-mTLR5-V5)	Amp^R	This study
pCJ801	pEF6-V5	protein expression plasmid for chicken TLR5 (pEF6-chTLR5-V5)	Amp^R	This study
pCJ1413	pEF6	Protein expression plasmid for chTLR5 without V5	Amp^R	This study
pCJ1414	pEF6-V5	protein expression plasmid for pig TLR5 (pEF6-piTLR5-V5)	Amp^R	This study
pCJ1406	pEF6-V5	protein expression plasmid for bovine TLR5 (pEF6-boTLR5-V5)	Amp^R	This study
pCJ1045	pEF6-V5	protein expression plasmid for chimeric mouse/human TLR5 (pEF6-m/hTLR5-V5)	Amp^R	This study
pCJ1040	pEF6-V5	protein expression plasmid for chimeric chicken/human TLR5 (pEF6-ch/hTLR5-V5)	Amp^R	This study
pCJ1044	pEF6-V5	protein expression plasmid for chimeric pig/human TLR5 (pEF6-pi/hTLR5-V5)	Amp^R	This study
pCJ1407	pEF6-V5	protein expression plasmid for chimeric bovine/human TLR5 (pEF6-bo/hTLR5-V5)	Amp^R	This study
	pET28a	Protein expression vector, T7 *lac* promoter, 6xHis tag, T7 tag	Km^R	Novagen
pCJ375	pET28a	*S. enterica* serovar Typhimurium LT2 *fliC* cloned into pET28a via BamHI and XhoI	Km^R	S. K. Lee and C. Josenhans, unpublished

Amp^R : ampicillin resistance, Km^R : kanamycin resistance

Table S3: Oligonucleotides used for gene amplification and cloning.

Gene	Primers	Sequence (5'-3')[a]	T_m (°C)	Reference
SalTy LT2 *fliC*	SalLT2FliCBamHI(F)	CGGGATCCATGGCACAAGTCATTAATAC	49	S. K. Lee and C. Josenhans, unpublished
	SalT2FliCEcoRI(R)	CGGAATTCCGCAGTAAAGAGAGGACG	51	S. K. Lee and C. Josenhans, unpublished
chTLR5	chTLR5_F1	AAAGGTACCGAGTCCGGATCCATGATGTTACAATCAACGGCTAATAATTG	65	This study
	chTLR5_R1	AAAAGCGGCCGCCGTGTGAGACTGTCGCTATAGTTTG	69	This study
	chTLR5_R2	AGAACTTAAGTGTCTGCTGGAG	55	This study
mTLR5	mTLR5_F2	AAAAGGTACCGCCGCCACCATGGCATGTCAACTTGACTTG	65	This study
	mTLR5_R2	AAAAGGTACCGCCGCCACCATGGCATGTCAACTTGACTTG	51	This study
	mTLR5_R4	GGAAAACTTAAAGAGACCCATGGCTTC	57	This study
piTLR5	piTLR5_F1	AAAAGGTACCGAGTCCGGATCCATGGGAGACTGCCTGGTCCTG	59	This study
	piTLR5_F2	ACCAAGTACATGGGAGTCCGGATCCATGGGAGACTGCCTGGTC	53	This study
	piTLR5_R1	AAAAGCGGCCGCCGGAGATGGTCACGCTTTGCAAC	67	This study
	piTLR5_R2	AAAACTTAAGGGACTCTAAGAC	55	This study
boTLR5	boTLR5_BstXI_F	AAACCAAGTACATGGGCCGCCACCATGGGAGACTGCCTTGA	51	This study
	boTLR5_NotI_R2	AAAGCGGCCGCCGGAGATGGTGGTTACATTTT	51	This study
	boTLR5_BsaI_R	GGAAAACTTAAAGAGACCTAAAACTTC	51	This study
hMyD88	hMyD88_F1	TAAGAAGGACCAGCAGAGCC	59	S. K. Lee and C. Josenhans, unpublished
	hMyD88_R1	CATGTAGTCCAGCAACAGCC	59	S. K. Lee and C. Josenhans, unpublished

[a]restriction sites in oligonucleotides are underlined; T_m: annealing temperature.

Table S4: Oligonucleotides used for sequencing.

Gene	Primers	Sequence (5'-3')[a]	T_m (°C)	Reference
	pEF6_R2	ACAGATGGCTGGCAACTAGAAG	61	C. Josenhans, unpublished
hTLR5	hTLR5_2025_R	CTGGGCTGTCTTATAACAG	56	This study
chTLR5	chTLR5_F1	AAAGGTACCGAGTCCGGATCCATG ATGTTACATCAACGGCTAATAATTG	65	This study
	chTLR5_R2	AGAACTTAAGTGTCTGCTGGAG	55	This study
piTLR5	piTLR5_F1	AAAAGGTACCGAGTCCGGATCCAT GGGAGACTGCCTGGTCCTG	59	This study
	piTLR5_R2	AAAACTTAAGGGACTCTAAGAC	55	This study
boTLR5	boTLR5_738_F	AGCAATGCCATCAATGGGAG	55	This study
	boTLR5_1438_F	TTGGAGAAAATATGTTGCAGC	53	This study
	pUNO1_hEF1-HTLV_F	TCCCTTGGAGCCTACCTA	51	This study
	pUNO1_SV40_R	ATTGCAGCTTATAATGGTTAC	51	This study
Sal_fliC	FSa1	CAAGTCATTAATACMAACAGCC	55	(Dauga *et al.*, 1998)
	rFSa1	TTAACGCAGTAAAGAGAGGAC	55	(Dauga *et al.*, 1998)
	rFSa1_2	CGTAGCCGTATCGAAGAT	49	This study
Sal_fljB	rFSa2	CAAGTAATCAACACTAACAGTC	58	(Dauga *et al.*, 1998)
	rFSa2	TTAACGTAACAGAGACAGCAC	58	(Dauga *et al.*, 1998)
	rFSa1_3	CAACCTGTCTGAAGCGCGTAG	61	This study

Table S5: Oligonucleotides used for site-directed mutagenesis.

Gene	Primers	Sequence (5'-3')[a]	T_m (°C)	Reference
Insertion of stop codon upstream of V5 tag	pEF6_noV5_F	CCGCTCGAGTCTATAGGGCCCGCGGTTCG	>81	This study
	pEF6_noV5_R	CGAACCGCGGGCCCTATAGACTCGAGCGG	>81	This study
Change N to A in chTLR5 (aa 744)	chTLR5_Ala_F	GCCTGGGGAAGAACATATCGCCAATATTCGTGATGCTATTTGG	>81	This study
	chTLR5_Ala_R	CCAAATAGCATCACGAATATTGGCGATATGTTCTTCCCCAGGC	>81	This study

aa: amino acid

Table S6: Antibodies used in this study.

Antibody[a]	Application	Description/Dilution	Source
α-V5	WB, IF	Mouse, monoclonal/ 1:5,000 (WB), 1:500 (IF)	Invitrogen, R960-25
α-actin [a]	WB	Mouse, monoclonal/1:30,000	Chemicon, MAB1501
α-GAPDH [a]	WB	Rabbit, monoclonal/1:1,000	Cell Signaling, 14C10
α-p38 [a]	WB	Rabbit, polyclonal/1:1,000	Cell Signaling, 9212
α-P-p38 [a] (Thr180/Tyr182)	WB	Rabbit, polyclonal/1:1,000	Cell Signaling, 9211
α-IRAK-I [a]	WB	Rabbit, polyclonal/1:500	Cell Signaling, 4359
α-IRAK-M [a]	WB	Rabbit 1:1,000	Abcam, ab16534
α-MyD88 [a]	WB	Rabbit, polyclonal/1:1,000	Abcam, ab2068
α-*E. coli* flagellin	WB	Mouse, monoclonal/1:500	Hölzel Diagnostika, 15D8
Goat α-rabbit IgG (H+L)	WB	Peroxidase-conjugated, AffiniPure/1:10,000	Dianova/Jackson
Goat α-mouse IgG (H+L)	WB	Peroxidase-conjugated, AffiniPure $F(ab')_2$ /1:10,000	Dianova/Jackson
Goat α-mouse Alexa Fluor™ 488	IF	1:5,000	Molecular Probes/ ThermoScientific

[a] detected species: human

WB: Western Blot

IF: Immunofluorescence

Table S7: Oligonucleotides used for RT-PCR of chicken (*Gallus gallus*) genes.

Gene	Primers	Sequence (5'-3')[a]	T_m (°C)	Reference[a]
chGAPDH	chGAPDH_F2	AGGGTGGTGCTAAGCGTGTT	59	(Faber *et al.*, 2016)
	chGAPDH_R2	AAGGGTGCCAGGCAGTTG	59	(Faber *et al.*, 2016)
chIL-CXCLi2 (chIL-8)	chIL-8_F1	CTGGCCCTCCTCCTGGTTTC	59	(Faber *et al.*, 2016)
	chIL-8_R1	TGGCGTCAGCTTCACATCTTG	59	(Faber *et al.*, 2016)
chIL-1β	chIL-1_F1	CTGAGTCATGCATCGTTTATGTTT C	59	(Faber *et al.*, 2016)
	chIL-1_R1	AAATACCTCCACCCCGACAAG	59	(Faber *et al.*, 2016)

[a] primers were designed and generously provided by Bernd Kaspers (LMU Munich)

Supplementary Reference List

1. Dauga, C., Zabrovskaia, A., and Grimont, P.A. (1998) Restriction fragment length polymorphism analysis of some flagellin genes of *Salmonella enterica*. *J Clin Microbiol* **36**: 2835-2843.

2. Faber, E., Gripp, E., Maurischat, S., Kaspers, B., Tedin, K., Menz, S. *et al.* (2016) Novel Immunomodulatory Flagellin-Like Protein FlaC in *Campylobacter jejuni* and Other *Campylobacterales*. *mSphere* **1**.

3. Gripp, E., Hlahla, D., Didelot, X., Kops, F., Maurischat, S., Tedin, K. *et al.* (2011) Closely related *Campylobacter jejuni* strains from different sources reveal a generalist rather than a specialist lifestyle. *BMC Genomics* **12:** 584-12.

4. Huh, J.W., Shibata, T., Hwang, M., Kwon, E.H., Jang, M.S., Fukui, R. *et al.* (2014) UNC93B1 is essential for the plasma membrane localization and signaling of Toll-like receptor 5. *Proc Natl Acad Sci U S A* **111**: 7072-7077.

5. Kim, J., Huh, J., Hwang, M., Kwon, E.H., Jung, D.J., Brinkmann, M.M. *et al.* (2013) Acidic amino acid residues in the juxtamembrane region of the nucleotide-sensing TLRs are important for UNC93B1 binding and signaling. *J Immunol* **190**: 5287-5295.

6. Larkin, M.A., Blackshields, G., Brown, N.P., Chenna, R., McGettigan, P.A., McWilliam, H. *et al.* (2007) Clustal W and Clustal X version 2.0. *Bioinformatics* **23**: 2947-2948.

7. Lee, S.K., Stack, A., Katzowitsch, E., Aizawa, S.I., Suerbaum, S., and Josenhans, C. (2003) *Helicobacter pylori* flagellins have very low intrinsic activity to stimulate human gastric epithelial cells via TLR5. *Microbes Infect* **5**: 1345-1356.

8. Nicholas, K.B., Nicholas H.B.J., and Deerfield, D.W.I. (1997) GeneDoc: Analysis and Visualization of Genetic Variation. *EMBNEW NEWS* **4**: 14.

9. Sievers, F., Wilm, A., Dineen, D., Gibson, T.J., Karplus, K., Li, W. *et al.* (2011) Fast, scalable generation of high-quality protein multiple sequence alignments using Clustal Omega. *Mol Syst Biol* **7**: 539.

10. Smith, M.F., Jr., Mitchell, A., Li, G., Ding, S., Fitzmaurice, A.M., Ryan, K. *et al.* (2003) Toll-like receptor (TLR) 2 and TLR5, but not TLR4, are required for *Helicobacter pylori*-induced NF-kappa B activation and chemokine expression by epithelial cells. *J Biol Chem* **278**: 32552-32560.

11. Tamura, K., Peterson, D., Peterson, N., Stecher, G., Nei, M., and Kumar, S. (2011) MEGA5: molecular evolutionary genetics analysis using maximum likelihood, evolutionary distance, and maximum parsimony methods. *Mol Biol Evol* **28**: 2731-2739.

12. Xu, Y., Tao, X., Shen, B., Horng, T., Medzhitov, R., Manley, J.L., and Tong, L. (2000) Structural basis for signal transduction by the Toll/interleukin-1 receptor domains. *Nature* **408**: 111-115.
